# CD74 Blockade Disrupts Endothelial Migrasome Signaling to Prevent Inflammatory Macrophage Differentiation and Inhibit Atherosclerotic Progression

**DOI:** 10.1002/advs.202502838

**Published:** 2025-06-23

**Authors:** Kangnan Zhang, Jiong Chen, Zhenhua Zhu, Hong Hu, Qinghui Zhang, Rongrong Jia, Na Wang, Shihao Xiang, Yong Zhou, Yuehong Wang, Ling Xu

**Affiliations:** ^1^ Clinical Research Institue Shanghai General Hospital Shanghai Jiao tong University School of Medicine Shanghai 200080 China; ^2^ Department of Gastroenterology Tongren Hospital Shanghai Jiao tong University School of Medicine Shanghai 200336 China; ^3^ Key Laboratory for Translational Research and Innovative Therapeutics of Gastrointestinal Oncology Tongren Hospital Shanghai Jiao Tong University School of Medicine Shanghai 200336 China; ^4^ Key Laboratory of Cell Differentiation and Apoptosis of Chinese Ministry of Education Department of Pathophysiology Shanghai Jiao Tong University School of Medicine (SJTU‐SM) Shanghai 200001 China; ^5^ Department of Clinical laboratory Tongren Hospital Shanghai Jiao Tong University School of Medicine Shanghai 200336 China; ^6^ Department of Cardiology 2nd Affiliated Hospital of Harbin Medical University Harbin 150001 China; ^7^ National Key Laboratory of Frigid Zone Cardiovascular Diseases; The Key Laboratory of Myocardial Ischemia Chinese Ministry of Education Harbin 150001 China

**Keywords:** APP‐CD74, atherosclerotic, endothelial cell, macrophage, migrasome

## Abstract

Endothelial dysfunction and abnormal activation of the monocyte‐macrophage system form a critical loop in atherosclerosis. The role of migrasomes in endothelial‐immune interactions remains unclear. This study explores migrasome evolution in the atherosclerotic (AS) microenvironment, highlighting their function as amplifiers in the inflammatory cascade. Through analysis of ApoE^−/‐^ mouse models and single‐cell multi‐omics data, migrasome activity is mapped using Gene Set Variation Analysis (GSVA) algorithms. Co‐culture systems and anti‐Cluster of differentiation 74 (CD74) blocking experiments are employed to investigate immune‐metabolic reprogramming triggered by migrasome cargo signaling. Advanced imaging and functional studies demonstrated that the interaction between amyloid protein precursor (APP) ligands on endothelial cells and CD74 receptors on macrophages triggers endothelial cells to produce more migrasomes. The clinical relevance of these findings is confirmed through CD74 blocking experiments, which effectively disrupted migrasome‐mediated signaling and attenuated atherosclerotic progression. Importantly, migrasome content is positively correlated with the severity of atherosclerosis. These results fundamentally challenge existing paradigms of intercellular communication by establishing migrasomes as dual‐functional entities – serving both as biomarkers of endothelial stress and molecular drivers of immune microenvironment deterioration. The discovery of the “migrasome‐APP‐CD74” signaling network opens new avenues for developing organelle‐targeted therapies to interrupt the vicious cycle of vascular inflammation.

## Graphical abstract

Migrasomes play a crucial role in endothelial‐immune interactions in atherosclerosis. This study identifies migrasomes as amplifiers in the inflammatory cascade, driving immune‐metabolic reprogramming via “migrasome‐APP‐CD74” signaling. Blocking CD74 disrupts migrasome‐mediated signaling, attenuating atherosclerosis progression. Migrasome content correlates with atherosclerosis severity, providing potential therapeutic targets.

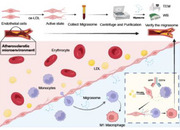

## 1 Introduction

Atherosclerosis (AS), as the ultimate manifestation of chronic vascular wall inflammation, is characterized by a cascade of pathological changes including lipid core formation, collagen matrix remodeling, and immune cell infiltration.^[^ ^1^, ^2^, ^3^ ^]^ Although lipid‐lowering strategies, such as statins, significantly reduce the risk of plaque rupture,^[^ ^4^ ^]^ residual inflammatory risk remains a critical driver of acute cardiovascular events.^[^ ^5^, ^6^, ^7^ ^]^ Atherosclerosis is closely associated with chronic systemic inflammation and hyperlipidemia, often accompanied by other metabolic abnormalities.^[^ ^8^ ^]^ Contemporary research has increasingly revealed that endothelial cell (EC) dysfunction and abnormal activation of the monocyte‐macrophage system represent the pathological core, with these processes driving a self‐amplifying chronic inflammatory microenvironment through complex molecular interactions ^[^ ^9^ ^]^ While much of the current research focuses on the roles of immune cells and endothelial cells (ECs) in atherosclerosis, the regulatory network and molecular features of ECs in atherosclerotic development remain incompletely understood.

The breakthrough of single‐cell transcriptomics has provided a novel paradigm for deciphering the cellular interaction networks in atherosclerosis. Studies have shown that the lineage plasticity of vascular smooth muscle cells,^[^ ^10^, ^11^ ^]^ the spatiotemporal heterogeneity of macrophage polarization phenotypes, and functional subtyping of endothelial populations all play critical roles in disease progression.^[^ ^10^, ^12^, ^13^, ^14^ ^]^ Notably, the capacity of endothelial cells to communicate across cells via extracellular vesicles (EVs) has gained increasing attention, especially the endothelial EVs responding to varying shear stress, which have been shown to regulate macrophage phenotype switching.^[^ ^15^, ^16^ ^]^ However, classic EVs, typically ranging from 100–200 nm in diameter, represent only one functional component of the endothelial secretome. Recently, migrasomes, a subcellular organelle with a unique biosynthetic origin, have been formally defined. These structures, with diameters ranging from 0.5 to 3 µm, are actively released through “filamentous structures” at the end of cytoplasmic migration tracks. Migrasomes have shown indispensable biological functions in embryonic development and tissue regeneration,^[^ ^17^ ^]^ yet their role in vascular disease remains unexplored.

Cluster of differentiation 74 (CD74), a transmembrane signal adaptor molecule, plays a dual role in modulating inflammation. It serves as a major histocompatibility complex (MHC) class II chaperone involved in antigen presentation, while also acting as a receptor for macrophage migration inhibitory factor (MIF), activating pro‐inflammatory pathways such as NF‐κB.^[^ ^18^, ^19^ ^]^ Clinical studies have confirmed a positive correlation between CD74 expression within atherosclerotic plaques and the severity of lesions.^[^ ^19^, ^20^ ^]^ However, the driving mechanisms of CD74 in endothelial‐immune interactions remain poorly understood, with key unresolved questions:^[^ ^21^, ^22^ ^]^ (1) Which endothelial subtypes secrete CD74 ligands? (2) Do specific organelles mediate the spatial delivery of these signaling molecules? (3) Can intervention in this pathway reverse disease progression?

This study integrates cross‐species single‐cell multi‐omics analysis with functional validation models: by analyzing the transcriptomes of human and mouse atherosclerotic endothelial cells, we first identified the Endo1 endothelial subpopulation as a functional hotspot for migrasome generation. We further elucidated how oxidized low density lipoprotein (ox‐LDL) induces endothelial cells to secrete migrasomes that carry amyloid protein precursor (APP) ligands, specifically activating macrophage CD74 receptors. Finally, through targeting antibody blockade models, we confirmed that inhibition of the APP‐CD74 axis effectively disrupts the “endothelial stress‐immune polarization” vicious cycle. These findings not only reveal a novel role for migrasomes as amplifiers of inflammation but also provide a theoretical framework for organelle‐based precision anti‐inflammatory strategies.

## 2 Results

### 2.1 Migrasomes are Highly Enriched in Atherosclerosis

**Figure 1:**
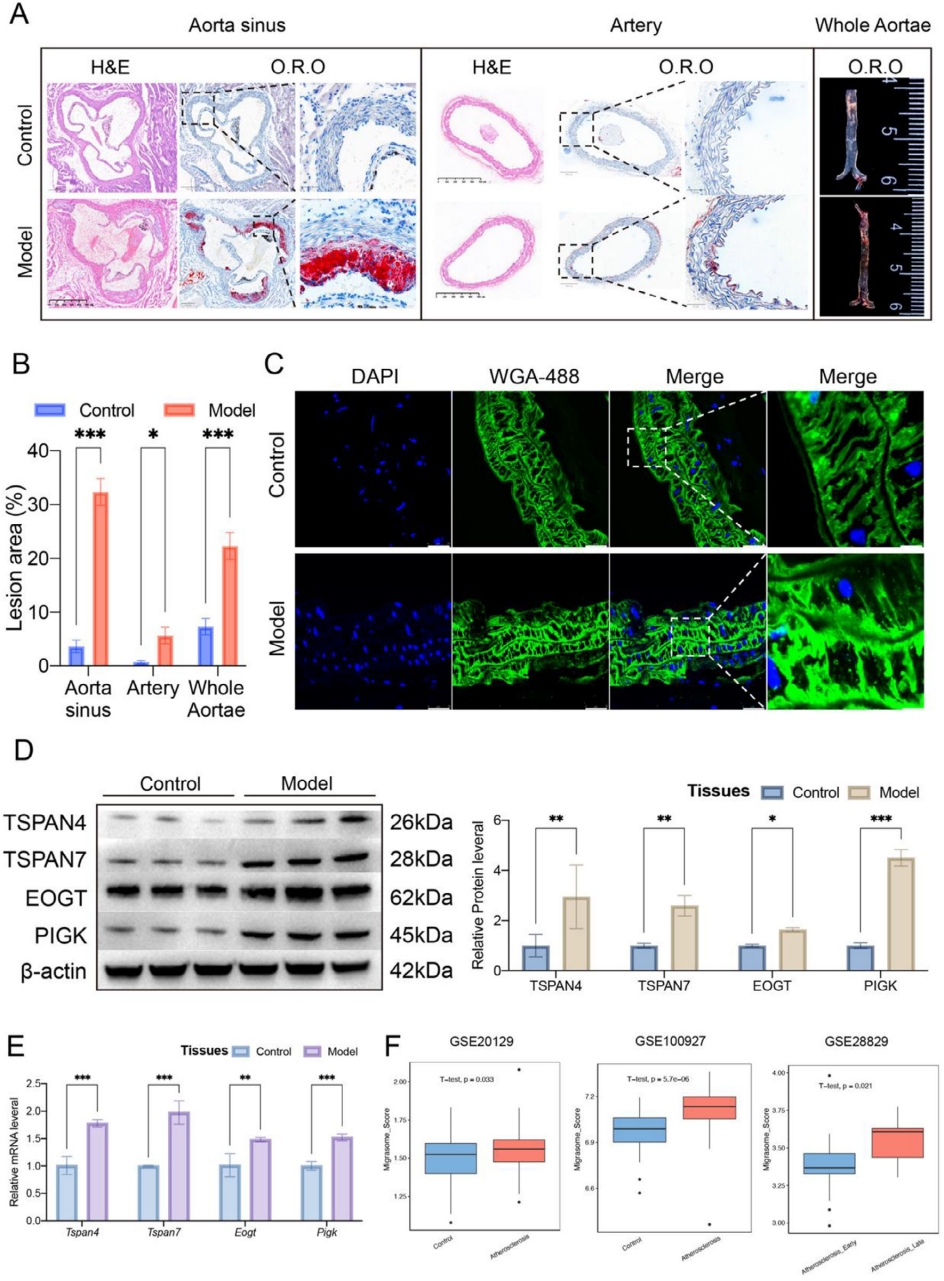
Migrasomes are enriched in atherosclerotic tissue. A,B) H&E staining and Oil Red O staining of murine aorta (Scale bars: 500 µm), with quantitative analysis of the atherosclerotic lesion/plaque area displayed in a bar chart (n = 5 mice/group). C) Immunofluorescence staining of murine aorta, showing migrasomes in green (WGA‐Alexa 488) and cell nuclei in blue (DAPI) (Scale bars: 500 µm, n = 5 mice/group). D) Western blot analysis of migrasome‐related marker proteins in the tissue, with quantification of protein expression shown in a bar chart (n = 3 mice/group). E) RT‐qPCR analysis of migrasome‐related gene mRNA expression in the tissue (n = 3 mice/group). F) Box plot showing Migrasome_Score expression levels in datasets GSE20129, GSE100927, and GSE28829. Data information: Data are expressed as mean ± SD. Two‐tailed unpaired Student's t‐tests, *p < 0.05; **p < 0.01; ***p < 0.001; ns, not significant.

We established an atherosclerosis model using ApoE^−/−^ mice, with C57BL/6 wild‐type mice serving as the control group. Oil Red O and HE staining were used to analyze the aorta of model mice. The results demonstrated that, compared to the control group, the model group showed significantly increased damage and plaque area in the aortic sinus, artery, and overall aorta (**Figure** **1**A, B), confirming the successful induction of atherosclerosis.

To further investigate, we performed fluorescence staining of aortic tissues using the migrasome‐specific dye WGA‐488. The results revealed a significant accumulation of migrasome‐like vesicles in the aortic tissue of the model group (Figure 1C), suggesting a potential role of migrasomes in atherosclerosis. To validate this observation, we analyzed the expression of migrasome‐related markers using Western Blot and RT‐PCR. The results showed a significant upregulation of migrasome‐associated genes, including TSPAN4, TSPAN7, EOGT, and PIGK, in the model group (Figure 1D,E), further supporting the high expression of migrasomes in atherosclerosis.

In parallel, to explore the mechanisms driving migrasome formation in the atherosclerotic environment, we performed in vitro experiments in endothelial cells. We treated HCAECs, MAECs and HUVECs with ox‐LDL to simulate the atherosclerotic conditions. The results from these experiments showed that the ox‐LDL‐treated endothelial cells generated numerous migrasomes, supporting the idea that the atherosclerotic environment stimulates migrasome formation (Figure 3; Figure S4, Supporting Information). These findings further validate the tissue‐level observations in Figure 1 and highlight the role of migrasomes in atherosclerosis.

Based on the expression profiles of key genes involved in migrasome formation (TSPAN4, TSPAN7, SGMS2, CERS5, COL4A3BP, and NDST1), we constructed a Migrosome_Score using the GSVA algorithm. Analysis of the GSE20129 dataset revealed that the expression of Migrosome_Score was significantly higher in peripheral blood samples from atherosclerotic patients compared to normal controls (Figure 1F). Further analysis of the GSE100927 dataset showed similar high levels of Migrosome_Score expression in atherosclerotic tissues, with a significant difference compared to normal control tissues (Figure 1F). Importantly, analysis of the GSE28829 dataset revealed that Migrosome_Score expression was significantly higher in late‐stage atherosclerotic patients than in early‐stage patients (Figure 1F), suggesting that migrasome expression is closely associated with the progression and severity of atherosclerosis.

In conclusion, this study is the first to demonstrate the significant enrichment of migrasomes in atherosclerosis, with their expression level positively correlating with the disease's severity.

### 2.2 Endothelial Cells Overexpress Migrasomes During Atherosclerosis

**Figure 2:**
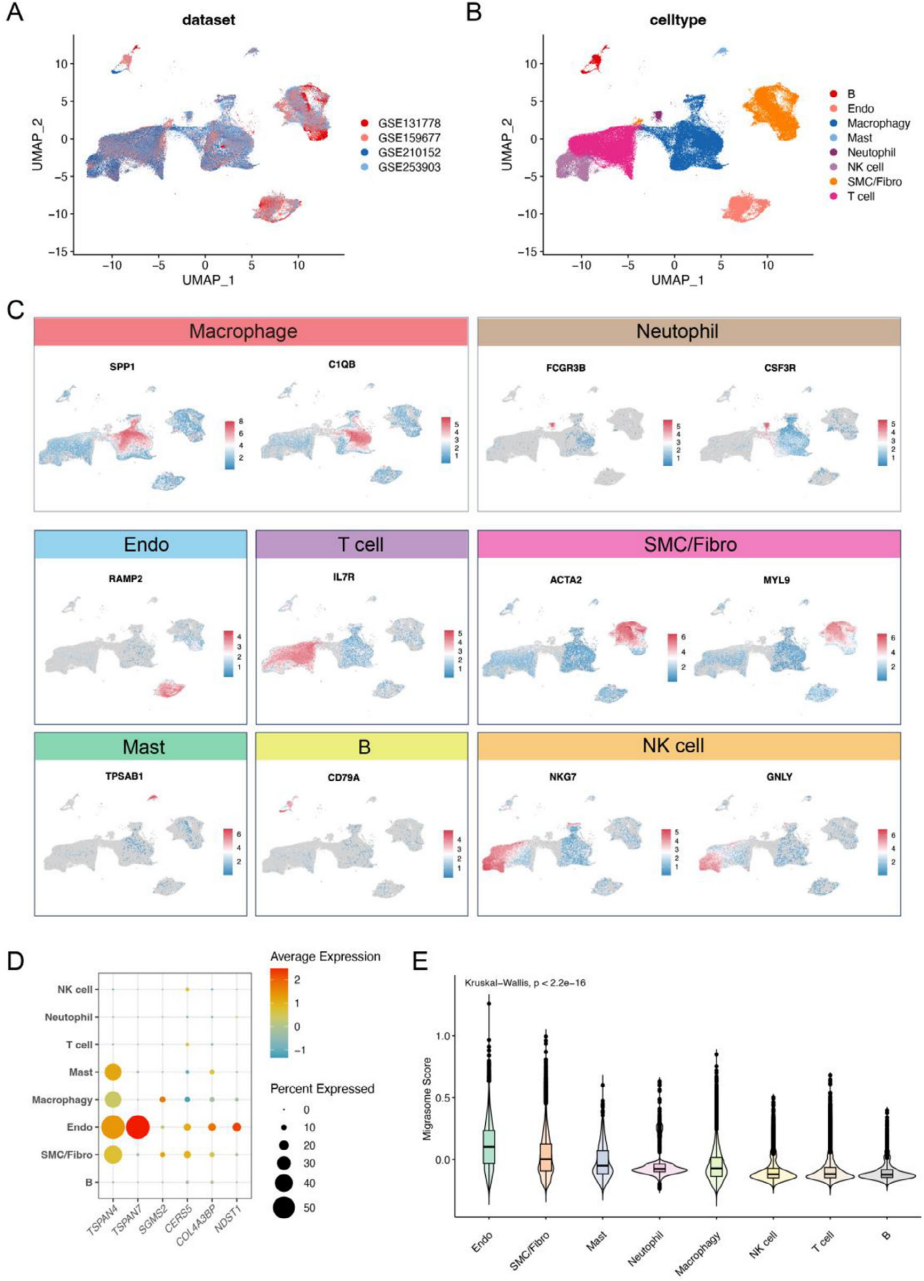
Single‐cell analysis of human atherosclerotic tissue. A) UMAP plot derived from four single‐cell datasets (GSE131778, GSE159677, GSE210152, and GSE253903) from atherosclerotic tissues, with each dataset represented in a different color. B) The four atherosclerotic datasets were clustered into 8 groups, with each cluster shown in a distinct color. Batch effects were corrected using the R package Harmony. C) Expression levels of known marker genes in unclassified cells from atherosclerotic tissue, displayed on a UMAP plot. D) Bubble plot showing the expression of migrasome‐related genes in different cell types, with the size of the bubbles representing the percentage of cells expressing each gene and the color indicating expression intensity. E) Box plot showing the expression levels of Migrasome_Score in different cell types.

To further investigate the relationship between the atherosclerotic tissue microenvironment and migrasomes, we integrated four single‐cell RNA sequencing (scRNA‐seq) datasets from human atherosclerotic patients: GSE131778, GSE159677, GSE210152, and GSE253903 (**Figure** **2**A). To correct for batch effects across the four datasets, we used the Harmony algorithm to integrate the atherosclerotic scRNA‐seq data. After batch effect correction, we generated a unified UMAP embedding space using the principal components corrected by Harmony, followed by a graph‐based clustering analysis to annotate each cluster. A total of eight major cell types were identified (Figure 2B), including SPP1^+^ C1QB^+^ macrophages; FCGR3B^+^ CSF3R^+^ neutrophils; RAMP2^+^ endothelial cells; IL7R^+^ T cells; ACTA2^+^ MYL9^+^ SMC/Fibroblasts; TPSAB1^+^ mast cells; CD79A^+^ B cells; and NKG7^+^ GNLY^+^ NK cells (Figure 2C; Figure S2A, Supporting Information).

In our analysis of migrasome marker gene expression (TSPAN4, TSPAN7, SGMS2, CERS5, COL4A3BP, and NDST1), we observed the highest expression levels of these genes in endothelial cells (Figure 2D). Further analysis using the Migrasome_Score showed that the migrasome content in endothelial cells was significantly higher than in other cell types (Figure 2E). To validate the conservation of this finding, we integrated the scRNA‐seq datasets of mouse atherosclerotic and normal tissues (GSE205931) (Figure S1A, Supporting Information). The results showed that the Migrasome_Score expression in atherosclerotic tissues was also significantly higher than in the control group (Figure S1B, Supporting Information). A total of five major cell types were identified from both atherosclerotic and normal tissues (Figure S1A, Supporting Information), including Ltb^+^ NK/T cells; C1qb^+^ macrophages; Pecam1^+^ endothelial cells; Dcn^+^ fibroblasts; and Myl9^+^ Myh11^+^ SMC cells (Figure S1C, Supporting Information). Similarly, migrasome‐related genes were expressed at higher levels in endothelial cells (Figure S1D, Supporting Information). Overall analysis of the Migrasome_Score further confirmed that endothelial cells exhibited the highest migrasome content (Figure S1E, Supporting Information). In summary, we validated the conservation of migrasome activity, with endothelial cells producing the most migrasomes during atherosclerosis in both human and mouse models.

### 2.3 Atherosclerotic Environment Stimulates Migrasome Formation in Endothelial Cells

**Figure 3:**
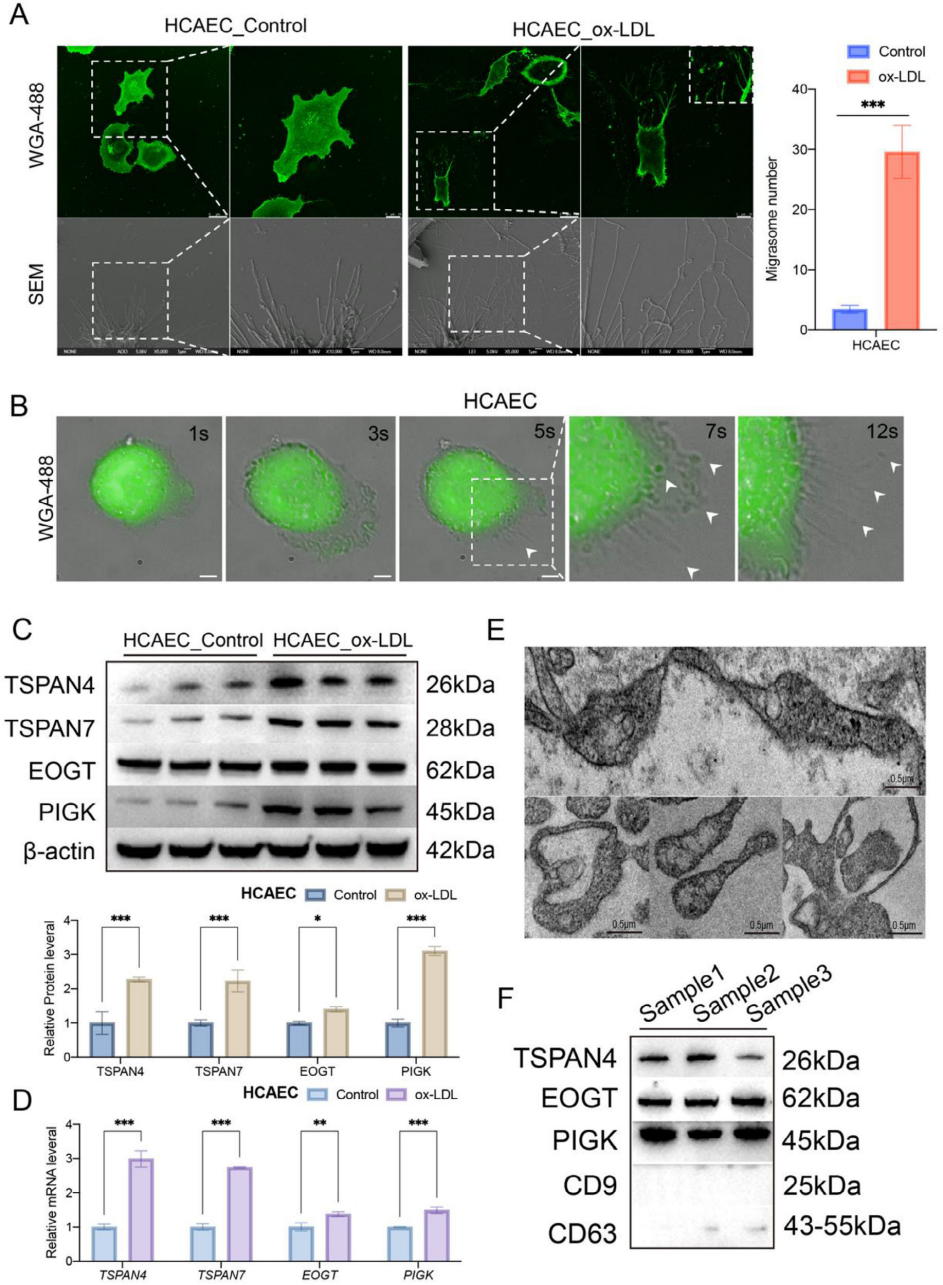
Human aortic endothelial cells generate migrasomes upon ox‐LDL stimulation. A) HCAEC cells exposed to ox‐LDL, stained with 1 µg/ml WGA‐Alexa 488 (Scale bars: 10 µm), and observed by confocal microscopy and SEM (Scale bars: 1 µm). Statistical analysis of migrasome numbers in control and ox‐LDL groups, normalized to the control group. Each group includes 50 cells. B) HCAEC cells exposed to ox‐LDL, stained with 1 µg/ml WGA‐Alexa 488, observed under high‐content imaging (Scale bars: 10 µm). C) Western blot analysis of migrasome marker expression in different cell lines, with a bar chart quantifying the results (*n* = 3 samples/group). D) RT‐qPCR analysis of migrasome‐related gene mRNA expression in cells (*n* = 3 samples/group). E) Observation of migrasome enrichment using scanning electron microscopy (SEM) (Scale bars: 0.5 µm). F) Western blot (WB) experiments validating marker expression in extracted migrasomes (*n* = 3 samples/group). Data information: Data are expressed as mean ± SD. Two‐tailed unpaired Student's t‐tests, *p < 0.05; **p < 0.01; ***p < 0.001; ns, not significant.

To investigate the impact of the atherosclerotic environment on migrasome generation in endothelial cells, we induced damage to HCAECs using oxidized low‐density lipoprotein (ox‐LDL), simulating an in vitro model of atherosclerosis. Scanning electron microscopy (SEM) revealed that ox‐LDL‐treated HCAECs exhibited numerous micron‐sized vesicles on their surface, with sizes ranging from approximately 0.5 to 2.0 microns (**Figure** **3**A). These vesicles closely resembled migrasomes in both size and morphology as previously reported by Yu et al., suggesting that they could be migrasomes. WGA‐488 staining further confirmed that, under ox‐LDL conditions, HCAECs generated abundant migrasomes, while no prominent migrasome formation was observed under normal culture conditions (Figure 3A).

To observe this process more directly, we utilized high‐content imaging to track migrasome formation in real‐time in ox‐LDL‐treated HCAECs. Dynamic time‐lapse imaging showed a gradual generation and release of migrasomes, which exhibited strong fluorescence signals upon WGA‐488 staining (Figure 3B). These findings suggest that the atherosclerotic environment significantly stimulates migrasome formation in endothelial cells, and that this process is observable in real‐time, supporting the hypothesis that migrasomes may serve as markers of endothelial cell activation. To further confirm that the vesicles generated were indeed migrasomes and not other types of vesicles, we performed Western blot (Figure 3C) and RT‐PCR (Figure 3D) analyses on HCAEC_Control and HCAEC_ox‐LDL groups. The results demonstrated a significant upregulation of migrasome‐associated markers (such as TSPAN4, TSPAN7, EOGT, and PIGK) in ox‐LDL‐treated HCAECs, validating that ox‐LDL treatment promotes migrasome generation. To further investigate the molecular mechanism by which ox‐LDL induces migrasome formation in vascular endothelial cells, we referred to the pathway proposed by Ding et al., which identifies the PI(4,5)P₂–Rab35 axis as a critical regulator of migrasome biogenesis.^[^ ^23^ ^]^ Their study demonstrated that PIP5K1A is initially recruited to nascent migrasome sites, where it catalyzes the localized production of PI(4,5)P₂. The resulting enrichment of PI(4,5)P₂ subsequently recruits Rab35 to the membrane, which in turn facilitates the accumulation of integrin ITGα5, promoting migrasome formation. This mechanism is reportedly conserved across various cell types.

Building on this theory, we performed a series of experiments to assess whether ox‐LDL‐induced migrasome formation in endothelial cells depends on the same pathway. Upon treatment with 100 µg mL^−1^ ox‐LDL, we observed clear co‐localization of PIP5K1A and WGA‐labeled migrasomes, suggesting that PIP5K1A is involved in early‐stage migrasome formation (Figure S3A, Supporting Information). Confocal imaging revealed that PI(4,5)P₂ and Rab35 were both enriched at the migrasome initiation site prior to the accumulation of ITGα5 (Figure S3B,C, Supporting Information). These observations suggest that the recruitment of PI(4,5)P₂ and Rab35 precedes ITGα5 during ox‐LDL‐induced migrasome formation. While our data demonstrated that both PI(4,5)P₂ and Rab35 temporally localize before ITGα5, the sequential relationship between PI(4,5)P₂ and Rab35 is supported by previous studies. Notably, Heo et al.^[^ ^24^ ^]^ reported that Rab35 membrane localization requires its C‐terminal polybasic cluster, which directly interacts with PI(4,5)P₂. Furthermore, Ding et al.^[^ ^23^ ^]^ showed that mutating this polybasic region (Rab35‐7A) disrupts Rab35 recruitment to membranes and abolishes migrasome formation. Together, these findings indicate that PI(4,5)P₂ facilitates the recruitment of Rab35, which subsequently promotes ITGα5 accumulation and migrasome biogenesis.

To functionally validate this pathway, we employed siRNA‐mediated knockdown of PIP5K1A and Rab35, and confirmed gene silencing by qPCR (Figure S3D, Supporting Information). Immunofluorescence imaging revealed that knockdown of either PIP5K1A or Rab35 significantly reduced the number of migrasomes formed in response to ox‐LDL stimulation, compared to the control group (Figure S3E, Supporting Information). These findings confirm that both PIP5K1A and Rab35 are indispensable for ox‐LDL‐induced migrasome formation.

This sequential recruitment pattern provides strong evidence that the PI(4,5)P₂–Rab35–ITGα5 axis mediates ox‐LDL‐induced migrasome formation in vascular endothelial cells.

For additional confirmation, we isolated the vesicles generated by HCAEC_ox‐LDL cells and examined their ultrastructure using transmission electron microscopy (TEM) (Figure 3E). TEM images revealed that these vesicles exhibited the typical morphological features of migrasomes, consistent with structures previously described in the literature. Subsequently, Western blot analysis confirmed that these vesicles were migrasomes, not exosomes, further validating our observations (Figure 3F). To investigate the broader effects of the atherosclerotic environment on endothelial cells, we conducted similar experiments in Mouse Aortic Endothelial Cells (MAECs) and Human Umbilical Vein Endothelial Cells (HUVECs). Under ox‐LDL treatment, both MAEC and HUVEC cells produced abundant migrasomes, which were clearly visible through WGA‐488 staining and SEM imaging (Figure S4A, Supporting Information). Additional Western blot (Figure S4B, C, Supporting Information) and RT‐PCR (Figure S4D, E, Supporting Information) analyses showed significant upregulation of migrasome‐associated markers (TSPAN4, TSPAN7, EOGT, and PIGK) in both MAEC_ox‐LDL and HUVEC_ox‐LDL cells.

In summary, the atherosclerotic environment significantly stimulates migrasome formation in both human and mouse endothelial cells. This finding suggests that migrasomes may not only serve as biomarkers of endothelial stress but could also play an important role in the initiation and progression of atherosclerosis.

### 2.4 Migrasomes Produced by Endo1 Endothelial Cells are Associated with Macrophage Polarization

**Figure 4:**
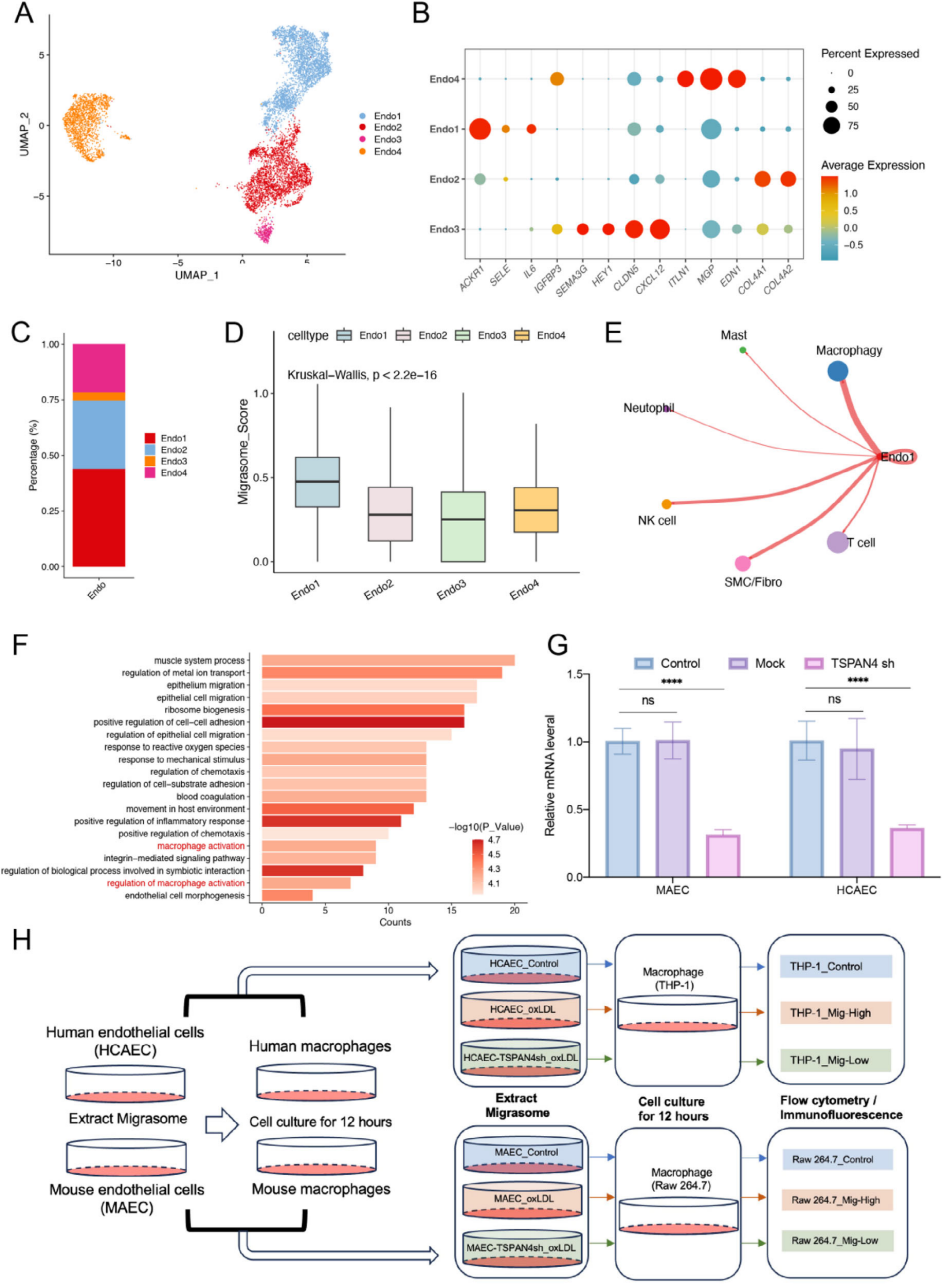
Single‐cell analysis of endothelial cells in human atherosclerotic tissue. A) Endothelial cells clustered into 4 groups, with each cluster shown in a different color. Batch effects were corrected using the R package Harmony. B) Dot plot showing the average expression of known markers in cell clusters, with the size of the dots representing the percentage of cells expressing each gene and the color indicating expression intensity. C) Pie chart showing the proportion of different types of endothelial cells in the dataset, with each cell type shown in a distinct color. D) Box plot showing Migrasome_Score expression levels in four different types of endothelial cells. E) Circular plot visualizing cell‐cell communication between Endo1 cells, T cells, SMC/Fibro cells, NK cells, neutrophils, Msat cells, and macrophages. The size of each circle corresponds to the number of cells in each cluster, and the line thickness indicates the weight of interactions. F) GO analysis of differentially expressed genes between Endo1_Mig‐High and Endo1_Mig‐Low groups. G) RT‐qPCR analysis of TSPAN4 mRNA expression levels in MAEC and HCAEC cells transfected with TSPAN4 shRNA or Mock, compared to untreated MAEC/HCAEC‐Control cells (*n* = 3 samples/group). H) Schematic illustrating the experimental workflow for studying the activation of macrophages by migrasomes derived from endothelial cells. Data information: Data are expressed as mean ± SD. Two‐tailed unpaired Student's t‐tests, *p < 0.05; **p < 0.01; ***p < 0.001; ns, not significant.

Analysis of the GSE28829 dataset revealed that, in patients with advanced atherosclerosis, the expression of Migrasome_Score was significantly higher compared to early‐stage patients (Figure 1E), suggesting that migrasome expression is closely linked to the progression of atherosclerosis. Furthermore, endothelial cells were identified as the predominant source of migrasomes in atherosclerotic tissues, indicating their critical role in disease progression. Previous studies have shown that endothelial cells in atherosclerosis can be classified into distinct subtypes based on specific characteristics. We integrated four human atherosclerotic single‐cell RNA sequencing (scRNA‐seq) datasets, categorizing endothelial cells into four subtypes using endothelial markers (**Figure** **4**A, B). By calculating the proportions of each subtype, we found that Endo1 endothelial cells constituted the largest subpopulation (Figure 4C). Further analysis revealed that Endo1 cells exhibited significantly higher Migrasome_Score expression compared to other subtypes (Figure 4D).

To further characterize the functional differences among these endothelial subtypes, we performed Gene Ontology (GO) enrichment analysis. Endo1 cells were significantly enriched in immune and inflammatory pathways, including targeting to membrane, interferon‐gamma‐mediated signaling pathway, and cellular response to tumor necrosis factor, suggesting a pro‐inflammatory phenotype that may underlie their elevated migrasome activity. Endo2 cells showed enrichment in extracellular matrix‐related pathways, such as cell‐substrate adhesion and extracellular structure organization, indicative of a role in maintaining vascular structure and cell‐matrix interactions. Endo3 cells were associated with core cellular processes, including ribosome biogenesis and mRNA catabolic processes, as well as moderate enrichment in interferon signaling, suggesting a transcriptionally active but less inflammatory phenotype compared to Endo1. In contrast, Endo4 cells were predominantly enriched in developmental pathways, such as cardiac chamber morphogenesis and endothelium development, implying a more quiescent or maturation‐associated state (Figure S5A, Supporting Information).

Given our earlier findings that ox‐LDL‐induced migrasome formation in endothelial cells depends on the PI(4,5)P₂–Rab35 axis, we examined the expression of these key pathway components across subtypes. PIP5K1A and ITGα5 were most highly expressed in Endo1, while Rab35 was highest in Endo3 and second highest in Endo1 (Figure S5B, Supporting Information). These results suggest that Endo1 is particularly primed to initiate migrasome formation via the PI(4,5)P₂–Rab35 pathway.

GO enrichment analysis demonstrated that high migrasome expression in Endo1 cells significantly activated macrophage‐related pathways (Figure 4F). Additionally, GSVA analysis showed substantial activation of macrophage activation pathways, such as “Macrophage activation” and “Regulation of macrophage activation,” in Endo1 cells (Figure S5C, D, Supporting Information). Intercellular interaction analysis further confirmed a strong interaction between Endo1 cells and macrophages in atherosclerotic samples (Figure 4E), while interactions between Endo2–4 cells and macrophages, as well as other cell types, are shown in Figure S2B (Supporting Information). Therefore, Endo1 endothelial cells are the main source of migrasomes in atherosclerosis, and these migrasomes likely contribute to macrophage activation, exacerbating disease progression.

**Figure 5:**
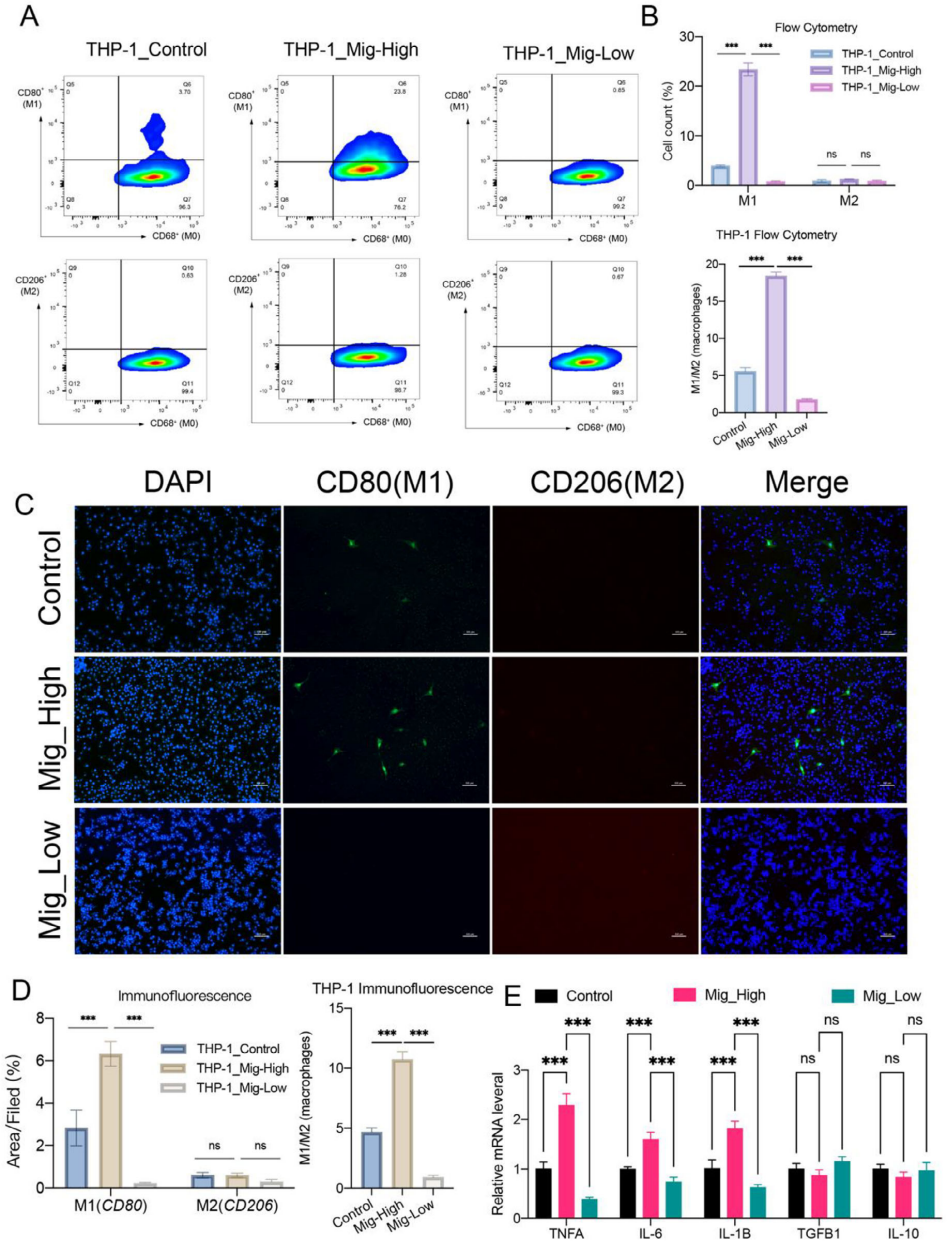
Validation of the effect of migrasomes generated by human endothelial cells on human‐derived macrophages. A) Flow cytometry analysis of macrophage differentiation. B) Statistical bar chart of macrophage differentiation from flow cytometry analysis (*n* = 3 samples/group). C) Immunofluorescence analysis of macrophage differentiation. Blue: DAPI; Green: M1 macrophages (CD80^+^); Red: M2 macrophages (CD206^+^) (Scale bars: 100 µm, *n* = 3 samples/group). D) Statistical bar chart showing the percentage of fluorescent regions in macrophage differentiation analyzed by immunofluorescence (*n* = 5 samples/group). E)qPCR analysis of cytokine expression in three groups of cells (*n =* 3 samples/group). Data information: Data are expressed as mean ± SD. One‐way analysis of variance (ANOVA) was used followed by Tukey's post hoc test to determine the statistical significance, *p < 0.05; **p < 0.01; ***p < 0.001; ns, not significant.

To investigate the role of migrasomes from high‐expressing Endo1 cells in macrophage activation, we stimulated MAEC and HCAEC cells with ox‐LDL to obtain MAEC_Mig‐High and HCAEC_Mig‐High cells (representing Endo1), and knocked down TSPAN4, a key gene involved in migrasome formation, to generate MAEC_Mig‐Low and HCAEC_Mig‐Low cells (Figure 4G). Control cells (MAEC‐Control and HCAEC‐Control) were left untreated. After 15 hours of culture, migrasomes were extracted from MAEC_Mig‐High, MAEC_Mig‐Low, and MAEC‐Control cells and co‐cultured with Raw264.7 cells; the same procedure was applied to HCAEC cells and co‐cultured with THP‐1 cells (experimental workflow shown in Figure 4H). Flow cytometry analysis at 12 hours demonstrated that migrasomes derived from HCAEC cells promoted the differentiation of human macrophages (THP‐1) into M1 macrophages (CD80⁺), while having a minimal effect on M2 macrophage differentiation (CD206⁺) (**Figure** **5**A,B). Similarly, migrasomes from MAEC cells promoted M1 macrophage differentiation (Nos2^+^) in mouse macrophages (Raw264.7), with little effect on M2 macrophage differentiation (Cd206^+^, Figure S6A, B, Supporting Information). Furthermore, by analyzing the M1/M2 ratio as an indicator of macrophage polarization, we observed that the Mig‐High group exhibited an enhanced inflammatory response, whereas the Mig‐Low group showed the lowest inflammatory activity (Figure 5B; Figure S6B, Supporting Information). Immunofluorescence results confirmed the flow cytometry findings (Figure 5C, D; Figure S6C, D, Supporting Information). To further validate the polarization status, we performed qPCR analysis of cytokine expression in macrophages from different groups. The Mig‐High group showed significantly elevated expression of pro‐inflammatory cytokines, including TNF‐α, IL‐6, and IL‐1β, while anti‐inflammatory cytokines such as TGF‐β1 and IL‐10 were slightly reduced without statistical significance (Figure 5E; Figure S6E, Supporting Information). These results indicate that migrasomes derived from Endo1 cells facilitate macrophage polarization toward the M1 phenotype.

In summary, Endo1 endothelial cells are the main source of migrasomes in atherosclerosis, and these migrasomes contribute to increased macrophage polarization toward M1, thereby exacerbating inflammatory infiltration in atherosclerosis.

### 2.5 Endothelial Cell and Macrophage Interaction Drives Migrasome Production and Atherosclerosis Progression

Our experiments indicate that migrasome‐high (Mig‐High) endothelial cells, akin to Endo1‐type cells, generate migrasomes that promote macrophage polarization towards the M1 phenotype, thereby advancing AS.

**Figure 6:**
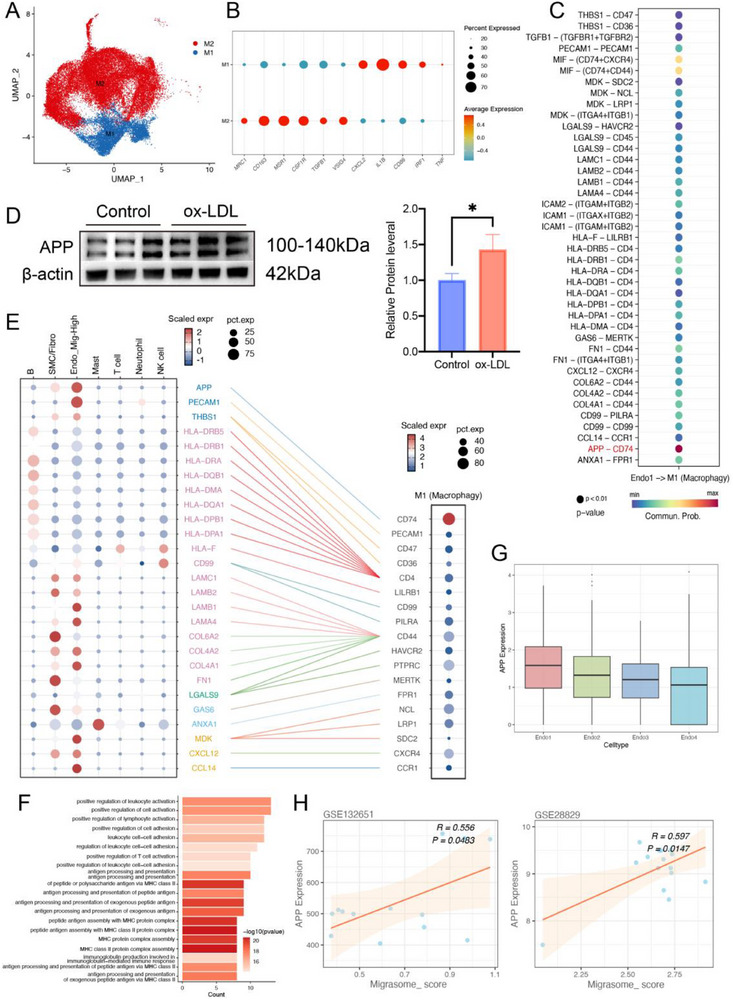
Ligand‐receptor analysis of interactions between Endo_Mig‐High and M1 macrophages. A) Macrophages were divided into two clusters, each represented by a different color. Batch effects were corrected using the R package Harmony. B) Dot plot showing the average expression of known markers within the cell clusters. The size of the dots represents the percentage of cells expressing each gene within the cluster. The intensity of the marker expression is displayed. C) Ligand‐receptor pairs between different cell types and M1 macrophages in atherosclerotic tissue. Dot color and size indicate the calculated communication probability and p‐value. D) Western blot analysis of APP expression in migrasomes derived from different cell lines, accompanied by a bar chart quantifying the results (*n* = 3 samples/group). E) Ligand‐receptor pairs between Endo_Mig‐High cells and M1 macrophages in atherosclerotic tissue. Dot color and size represent the communication probability and p‐value. F)GO analysis of differentially expressed genes encoding ligands. G) Box plot showing APP expression in four types of endothelial cells. H) Correlation analysis between APP expression and Migrasome_Score in GSE132651 and GSE28829 datasets. Data information: Data are expressed as mean ± SD. Two‐tailed unpaired Student's t‐tests, *p < 0.05; **p < 0.01; ***p < 0.001; ns, not significant.

To further elucidate the interplay between endothelial cells and macrophages and its impact on migrasome generation, we integrated scRNA‐seq datasets of macrophages, categorizing them into two distinct subsets based on specific molecular markers (**Figure** **6**A,B). Ligand‐receptor interaction analysis revealed that Endo1‐type endothelial cells primarily communicate with M1 macrophages via the APP–CD74 axis (Figure 6C). Specifically, Endo1 cells highly express APP and PECAM1, while M1 macrophages predominantly express CD74 (Figure 6E). Gene Ontology analysis of these ligands indicated activation of inflammatory processes such as “positive regulation of leukocyte activation” and “leukocyte cell‐cell adhesion” (Figure 6F). Notably, among all EC subtypes, Endo1 cells showed the highest expression of APP (Figure 6G), and APP levels positively correlated with migrasome scores in two independent atherosclerosis datasets (GSE132651 and GSE28829; r = 0.556, p = 0.048; r = 0.597, p = 0.015) (Figure 6H). To experimentally validate the role of endothelial APP in migrasome biogenesis and macrophage polarization, we conducted western blot analysis of migrasomes purified from endothelial cells treated with or without ox‐LDL. The results confirmed that APP was markedly enriched in migrasomes from ox‐LDL‐treated cells (Figure 6D). We further performed co‐culture experiments using human coronary artery endothelial cells (HCAECs) and THP‐1 cells. HCAECs were first transfected with either APP‐targeting siRNA (APP_si group) or control siRNA. After 24 hours of transfection, the cells were stimulated with ox‐LDL, for an additional 24 hours. Subsequently, equal numbers of THP‐1‐derived macrophages were added to the HCAEC cultures and co‐cultured for 12 hours under the same stimulation conditions. After co‐culture, the entire cell population, including both HCAECs and THP‐1‐derived macrophages, was harvested for protein extraction. Western blot analysis revealed that APP knockdown in HCAECs led to significantly reduced expression of migrasome markers (TSPAN4, TSPAN7, and EOGT), as well as M1 macrophage polarization marker CD80 and the CD74 receptor, compared to the control group. In contrast, the expression of the M2 marker CD206 was only slightly reduced and did not reach statistical significance, supporting a specific role of endothelial APP in promoting M1 polarization of macrophages (Figure S7A, Supporting Information).

**Figure 7:**
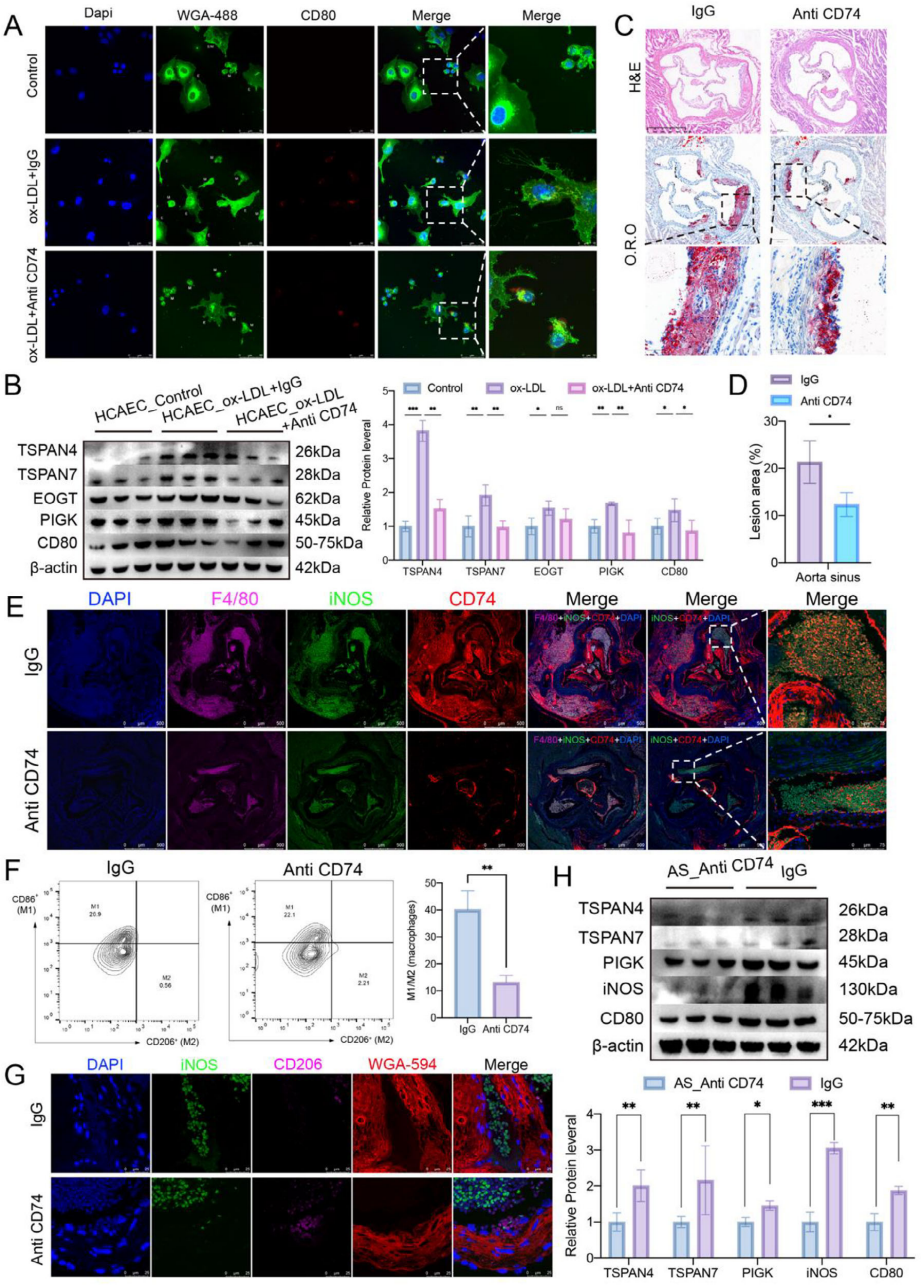
Impact of ligand‐receptor interactions between endothelial cells and macrophages on atherosclerosis. A) HCAEC cells treated with ox‐LDL and Anti‐CD74 for 12 hours, stained with 1 µg/ml WGA‐Alexa 488 and CD80, and observed under confocal microscopy (Scale bars: 50 µm). B) Western blot analysis of migrasomes and M1 macrophage‐related markers in HCAEC cells treated with ox‐LDL and Anti‐CD74, with statistical data presented in a bar chart (*n* = 3 samples/group). C) HE and O.R.O staining of aorta sinus from the AS and AS_Anti CD74 groups (*n* = 5 mice/group). D) Statistical bar chart of lesion area in the aorta sinus after staining in the AS and AS_Anti CD74 groups (*n* = 5 mice/group). E) Immunofluorescence staining of the aorta sinus from the AS and AS_Anti CD74 groups. Red: CD74 (labeling CD74 receptor); Magenta: F4/80 (labeling macrophages); Green: iNOS (labeling M1 macrophages); Blue: DAPI (nuclei) (*n* = 5 mice/group). F) Statistical bar chart showing the percentage of CD206⁺ (M2) and CD86⁺ (M1) macrophage‐positive fluorescent regions in arterial tissues, analyzed by flow cytometry (n = 5 samples/group). G) Immunofluorescence images showing M1 macrophages and migrasomes in Control, AS, and AS_Anti CD74 groups. Red: WGA‐594 (labeling migrasomes); Green: iNOS (labeling M1 macrophages); Magenta: CD206 (labeling M2 macrophages); Blue: DAPI (nuclei) (*n* = 5 mice/group). H) Western blot analysis of migrasomes and macrophage‐related markers in AS and AS_Anti CD74 groups, with statistical data presented in a bar chart (n = 3 mice/group). Data information: Data are expressed as mean ± SD. Two‐tailed unpaired Student's t‐tests (D,F,H) and one‐way analysis of variance (ANOVA) were used followed by Tukey's post hoc test to determine the statistical significance (B), *p < 0.05; **p < 0.01; ***p < 0.001; ns, not significant.

To further verify the functional involvement of the APP–CD74 axis, we co‐cultured THP‐1 macrophages with HCAECs under three conditions: Control, ox‐LDL+IgG, and ox‐LDL+Anti‐CD74. While ox‐LDL+IgG significantly promoted migrasome production and M1 differentiation, the Anti‐CD74 treatment markedly attenuated both processes (**Figure** **7**A,B). These findings confirm that endothelial‐derived APP facilitates migrasome‐mediated M1 polarization through interaction with macrophage CD74.

Single‐cell transcriptomic analysis revealed that macrophages are the most enriched CD74‐expressing cell population in the tissue (Figure S7B, Supporting Information). Therefore, upon treatment with Milatuzumab (an anti‐CD74 antibody) in mice, macrophages are the predominant cellular target of the antibody in the context of atherosclerosis. In vivo experiments utilizing ApoE^−/−^ mice on a high‐fat diet (HFD) to induce AS revealed that administration of Milatuzumab (anti‐CD74 antibody) every three days alleviated AS progression compared to the IgG group, as evidenced by histological staining (Figure 7C). Blood pressure measurements showed that the IgG group had a systolic blood pressure (SBP) of 134.0 ± 7.4 mmHg and a diastolic blood pressure (DBP) of 91.4 ± 2.7 mmHg, while the Anti‐CD74 group exhibited significantly lower SBP (121.2 ± 7.0 mmHg) and DBP (81.2 ± 3.5 mmHg) (Figure S7C, Supporting Information). Immunofluorescence staining further demonstrated reduced CD74 receptor expression on M1 macrophages and decreased macrophage polarization in the Anti‐CD74 group (Figure 7E). Single‐cell suspensions were prepared from arterial tissues and analyzed by flow cytometry. Dead cells were excluded using a viability dye (BV510), followed by sequential gating on CD45⁺ leukocytes and CD11b⁺ myeloid cells. Macrophages were identified as CD45⁺CD11b⁺F4/80⁺ cells. Flow cytometry analysis further confirmed a reduced proportion of CD86⁺ (M1) macrophages and a significantly decreased M1/M2 (CD86⁺/CD206⁺) ratio in the Anti‐CD74 group (13.08 ± 2.67) compared to the IgG control group (40.05 ± 6.85) (Figure 7F, S7D). Additionally, WGA‐594 labeling of migrasomes and iNOS staining of M1 macrophages revealed a significant reduction in both migrasomes and M1 macrophages in the Anti‐CD74 group compared to the IgG group. Conversely, the expression of the M2 macrophage marker CD206 was slightly increased in the Anti‐CD74 group, suggesting a potential shift toward an anti‐inflammatory phenotype (Figure 7G). Western blot analysis confirmed that migrasome and M1 macrophage markers were significantly lower in the Anti‐CD74 group (Figure 7H).

Functional changes related to AS symptoms were also assessed. Serum lipid profiles, including total cholesterol (TC), triglycerides (TG), HDL‐C, and LDL‐C, were measured in both groups. The Anti‐CD74 group showed a significant reduction in TG and LDL‐C levels, while HDL‐C levels were significantly elevated. Although TC levels did not show a statistically significant difference between the groups, the Anti‐CD74 group had lower TC levels compared to the IgG group (Figure S7E, Supporting Information). In addition, inflammatory markers, including LPS, TNF‐α, IL‐6, and IL‐10, were analyzed. The IgG group exhibited higher levels of LPS, TNF‐α, and IL‐6, with statistical significance, whereas IL‐10 levels were elevated in the Anti‐CD74 group, although the increase was not statistically significant (Figure S7F, Supporting Information).

Collectively, these findings indicate that the atherosclerotic microenvironment initiates the production of migrasomes by endothelial cells. Endothelial‐derived migrasomes interact with macrophages, promoting their polarization towards the M1 phenotype through the APP‐CD74 ligand‐receptor axis, thereby forming a positive feedback loop that accelerates AS progression. Intervention with anti‐CD74 antibodies effectively disrupts this interaction, attenuating the pathological progression of AS.

## 3 Discussion

Atherosclerosis plays a crucial role in cardiovascular and cerebrovascular diseases; however, much research is still needed to explore the mechanisms underlying atherosclerotic plaque formation, to benefit more patients.^[^ ^25^ ^]^ Currently, studies on the function of migrasomes are limited, and research on their role in cardiovascular diseases is virtually non‐existent. Here, we present the first study investigating the role of migrasomes in atherosclerosis. Our findings demonstrate that endothelial cells, by secreting migrasomes, promote macrophage polarization towards the M1 phenotype, thereby exacerbating inflammation and disease progression.

In this study, we first observed abundant migrasome‐like structures in atherosclerotic tissues. We hypothesize that, in addition to the roles currently known for migrasomes, such as mitochondrial quality control,^[^ ^26^ ^]^ substance transfer,^[^ ^27^ ^]^ signal integration,^[^ ^28^ ^]^ angiogenesis, and tumor metastasis,^[^ ^29^ ^]^ migrasomes may also participate in the atherosclerotic process. Our analysis revealed that migrasome expression is higher in patients with advanced atherosclerosis, suggesting that the presence of migrasomes correlates with the severity of the disease. Through experimental and single‐cell analyses, we classified endothelial cells into four types (Endo1, Endo2, Endo3, Endo4) and found that migrasomes are highly enriched in Endo1‐type endothelial cells. Furthermore, Endo1 endothelial cells predominantly interact with macrophages. This aligns with the findings of Yu et al., who showed that cholesterol metabolite 27HC mediates interactions between monocytes/macrophages and endothelial cells, promoting endothelial cell activation and accelerating atherosclerotic progression in hypercholesterolemia.^[^ ^30^ ^]^ This suggests that interactions between endothelial cells and macrophages indeed exacerbate atherosclerosis.

Further analysis revealed that migrasomes secreted by Endo1‐type endothelial cells can induce macrophage polarization toward the M1 phenotype through the interaction between the APP protein and the CD74 receptor. Many studies have highlighted the importance of CD74 in cardiovascular diseases, such as ischemic heart disease and cardiac hypertrophy.^[^ ^19^, ^31^ ^]^ However, our research provides a novel perspective on CD74's role in atherosclerosis, focusing on the migrasome‐mediated mechanism. We observed that the APP‐CD74 ligand‐receptor interaction between Endo1 endothelial cells and M1 macrophages forms a positive feedback loop, not only promoting the production of more migrasomes by endothelial cells but also driving further M1 polarization of macrophages.

Li et al. recently showed that EVs secreted by endothelial cells under physiological laminar flow conditions can reprogram M1 macrophages into M2 macrophages, providing protection against atherosclerosis.^[^ ^16^ ^]^ Our study on migrasomes also involves endothelial cell secretion, but why do we observe seemingly contradictory findings? We discovered that the EVs analyzed by Li et al. highly expressed “CD63” and “TSG101,” markers of exosomes,^[^ ^32^ ^]^ and had a diameter of approximately 100–150 nm, which matches the typical size of exosomes.^[^ ^33^ ^]^ Notably, Li et al. used a 0.22 µm filter to exclude larger vesicles during EV extraction, which means their study did not include migrasomes (which have a diameter of around 1 µm). Our analysis showed that migrasomes are significantly larger than exosomes, and through Western blotting, we confirmed that our migrasome extracts contained minimal exosome contamination. Furthermore, we rigorously classified endothelial cells into four subtypes and found that migrasomes produced by Endo1 endothelial cells play a significant role in promoting atherosclerotic progression. Therefore, both studies complement each other, shedding light on the multifaceted role of endothelial cells and providing new insights into the vesicles secreted by endothelial cells in atherosclerosis.

Despite observing migrasome expression in multiple cell types, we have not yet explored how migrasomes specifically influence the responses of different immune cell types. This opens up potential directions for future research. We also need to further validate the role of migrasomes in atherosclerosis in clinical settings and explore their therapeutic potential as a new target. As we continue to uncover the mechanisms of migrasome function, we could develop intervention strategies based on migrasomes to help halt the progression of atherosclerosis. Additionally, the interaction between migrasomes and immune cells opens up new avenues for immunotherapy, particularly in regulating inflammation and immune tolerance.

In conclusion, our study provides a fresh perspective on the pathogenesis of atherosclerosis, revealing the important role of migrasomes as potential regulatory factors in endothelial cell damage and inflammation. We believe that further research will not only clarify the specific role of migrasomes in atherosclerosis but also offer novel strategies for the clinical treatment of related diseases.

## 4 Experimental Section

### Bulk and Single‐Cell Transcriptomics

Single‐cell RNA sequencing datasets (scRNA‐seq) from human and murine atherosclerosis studies were obtained from the Gene Expression Omnibus (GEO; https://www.ncbi.nlm.nih.gov/gds). The human datasets comprised four cohorts (GSE131778, GSE253903, GSE159677, GSE210152), which were used for comparative analysis of Migrasome_ scores across different cell types. Murine data (GSE205931) served for cross‐species validation.

Bulk transcriptomic profiles (GSE20129, GSE28829, GSE100927) were used to assess differential Migrasome_ scores between atherosclerotic lesions and normal arterial tissues.

### Dimension Reduction and Clustering Analysis

The Seurat‐based analytical pipeline was employed to process four single‐cell atherosclerosis transcriptomic datasets. Feature scaling was performed using characteristics derived from FindVariableFeatures. Batch effect correction across samples was conducted using the RunHarmony method from the harmony package.^[^ ^34^ ^]^ Cellular clustering was performed using Seurat's FindClusters() function, which enables multi‐resolution population identification. This was coupled with dimensionality reduction via Uniform Manifold Approximation and Projection (UMAP), implemented using RunUMAP with harmony reduction parameters across dimensions 1–10 for visualization.

### Migrasome_Score Calculation

Migrasome_score was calculated using gene set variation analysis,^[^ ^35^ ^]^ employing a six‐gene signature established in recent studies: TSPAN4, TSPAN7, SGMS2(SMS2), CERS5, COL4A3BP(CERT), and NDST1.^[^ ^36^, ^37^ ^]^ Single‐cell data scoring followed published protocols^[^ ^38^ ^]^ (https://www.github.com/cssmillie/ulcerative_colitis), where cellular signature scores were determined by averaging scaled expression values across all signature genes.

### Cell‐to‐Cell Communication Analysis

Cell‐cell communication analysis was performed using CellChat v1.1.1.^[^ ^39^ ^]^ This was initiated by constructing CellChat objects based on cluster‐defined groupings. Pre‐processing was conducted using default parameters with the “CellChatDB.human” ligand‐receptor interaction database. Endo1 was designated as the primary sender cell population, and communication probabilities were calculated systematically using the computeCommunProb function for ligand‐receptor pairs and computeCommunProbPathway for signaling pathways. Resulting interaction networks were visualized through hierarchical, circular, and heatmap representations to delineate communication patterns.

### Identification and Functional Annotation of Differentially Expressed Genes

Differentially expressed genes (DEGs) between Endo1 and Endo4 subtypes were identified using Seurat's FindAllMarkers function with the parameters (min.pct = 0.2, logfc.threshold = 0.25, only.pos = TRUE). Statistical significance was determined using Wilcoxon rank‐sum testing, followed by Bonferroni‐adjusted p‐values. DEG visualization was performed using heatmaps to display log‐transformed scaled expression values. Functional annotation was carried out using the clusterProfiler v4.2.2 R package, applying cumulative hypergeometric distribution to calculate pathway enrichment probabilities based on observed gene counts.

### Cell Culture

THP‐1 cells were cultured in RPMI‐1640 medium supplemented with 10% fetal bovine serum (FBS), 0.05 mM β‐mercaptoethanol, and 1% penicillin/streptomycin (P/S). Raw264.7 cells, human umbilical venous endothelial cells (HUVECs), and murine aorta endothelial cells (MAECs) were cultured in DMEM containing 10% FBS and 1% P/S. Human coronary artery endothelial cells (HCAECs) were cultured in endothelial cell growth medium (Lonza, CC‐3202). All cells were maintained at 37 °C in a 5% CO₂ atmosphere. The cell lines were obtained from Promocell and the American Type Culture Collection (ATCC), and all cell cultures were confirmed to be mycoplasma‐free.

### Animal Experiments

Six‐week‐old male C57BL/6 ApoE^−/−^ mice were used for the experiments. The study was approved by the Ethics Committee of Shanghai Tongren Hospital (approval number: A2023‐119‐01). Animal procedures were performed in accordance with the Guidelines for the Care and Use of Laboratory Animals. Mice were housed in a specific pathogen‐free room, with a 12‐hour light/dark cycle at a constant temperature of 25±1 °C, and allowed free access to food and water. For atherosclerosis (AS) modeling, ApoE^−/−^ mice were fed a high‐fat diet (HFD) (XiangTong Biological Co., XT108C) for 12 weeks. To investigate the role of CD74 signaling in vascular disease, experimental mice were treated with Milatuzumab (anti‐CD74 antibody) (15 mg/kg, once every three days, MedChemExpress, HY‐P99731) to block CD74 expression. Control group mice were injected with InVivoMAb polyclonal Armenian hamster IgG (10 mg/kg, once every three days, Bio X Cell, BXC‐BE0091). Following treatment, mice were sacrificed, and tissue samples were collected for further analysis. Blood was then collected, and plasma was isolated by centrifugation at 3000 × g for 10 minutes at 4 °C. Total cholesterol (TC), triglycerides (TG), high‐density lipoprotein cholesterol (HDL‐C), and low‐density lipoprotein cholesterol (LDL‐C) levels were measured using commercial assay kits purchased from Nanjing Jiancheng Bioengineering Institute (Nanjing, China). Additionally, serum concentrations of cytokines, including lipopolysaccharide (LPS), tumor necrosis factor‐α (TNF‐α), interleukin‐6 (IL‐6), and interleukin‐10 (IL‐10), were quantified using enzyme‐linked immunosorbent assay (ELISA) kits obtained from Shanghai Hengyuan Biotechnology Co., Ltd. (Shanghai, China).

### Blood Pressure Measurement

Each mouse was placed in a plastic restrainer and allowed to rest for more than 10 minutes as part of an acclimatization procedure. Following this acclimatization period, blood pressure measurements were recorded. The parameters measured included systolic blood pressure (SBP) and diastolic blood pressure (DBP). Blood pressure data were collected using a non‐invasive tail‐cuff blood pressure monitor (MOORLAB NIBP, MOOR).

### RNA Interference, RNA Isolation, and Real‐Time PCR

Gene silencing was accomplished using shRNA constructs targeting sequences listed in Table S1 (Supporting Information), with scrambled shRNA serving as the negative control. Gene silencing was achieved using siRNA targeting the specific gene sequences listed in Table S2 (Supporting Information), with a non‐targeting siRNA used as the negative control. Total RNA was isolated from cells using Trizol reagent (Invitrogen, Shanghai), followed by cDNA synthesis with the Promega Reverse Transcription System (Madison, WI) using Oligo dT primers. Quantitative PCR analysis was performed on a Roche LightCycler 480 (Switzerland) using SYBR Green Premix Ex Taq (Takara, Japan), with the following thermal cycling conditions: initial denaturation at 95 °C for 5 minutes, followed by 50 cycles of 95 °C for 5 seconds and 60 °C for 30 seconds. GAPDH mRNA was used for normalization, and fold‐change calculations were performed using the 2^−ΔΔCt method. Primer sequences for target amplification were provided in Tables S3, S4 (Supporting Information).

### Western Blot

Total protein was extracted using a Total Protein Extraction Kit (KGP200, KeyGen BioTECH, China), and protein concentrations were determined using a BCA Protein Assay Kit (KGPBCA, KeyGen BioTECH, China). The samples were separated by 10% SDS‐PAGE and transferred to a PVDF membrane. The following primary antibodies were used: β‐actin (1:1000, sc‐47778, Santa Cruz Biotechnology, USA), EOGT (1:1000, ab190693, Abcam, USA), TSPAN4 (1:1000, ab181995, Abcam, USA), TSPAN7 (1:1000, 18695‐1‐AP, Proteintech, USA), PIGK (1:1000, ab201693, Abcam, USA), CD9 (1:1000, sc‐13118, Santa Cruz Biotechnology, USA), and CD63 (1:1000, sc‐5275, Santa Cruz Biotechnology, USA).

### Confocal Imaging

Cells were cultured in 35 mm confocal dishes for 10–12 hours, followed by fixation with 4% paraformaldehyde. Membranes were stained with WGA488 (1 µg/ml, W11261, Life Technologies) for 15 minutes (extended to 2 µg/ml for 30 minutes in tissue sections). Images were acquired using a NIKON A1RSiHD25 confocal system at a resolution of 1024 × 1024 pixels. For live‐cell imaging, dishes were pre‐coated with fibronectin and cells were cultured for 6 hours prior to imaging. Live‐cell recordings were performed under physiological conditions (37 °C, 5% CO2) using the NIKON A1 microscope, maintaining identical resolution.

### Migrasome Purification

Migrasome isolation was performed via iodixanol density‐gradient centrifugation (Optiprep kit, LYSISO1, Sigma‐Aldrich), using sequential centrifugation steps: initial clarification at 1000 g for 5 minutes at 4 °C to remove cell bodies, followed by debris elimination at 4000 g for 20 minutes at 4 °C. The crude migrasome pellet, obtained from 20000 g centrifugation for 20 minutes at 4 °C, was resuspended and lysed using Sigma‐Aldrich extraction buffer. Ultracentrifugation was then performed at 150000 g for 4 hours at 4 °C in an Optiprep gradient (3%, 5%, 8%, 12%, 16%, 19%, 22.5%, 27%). Gradient fractions were diluted in PBS (500 µL), pelleted at 20000 g for 30 minutes at 4 °C, and washed with PBS through centrifugation at 2000 g for 10 minutes at 4 °C. The final migrasome collection was obtained by pelleting at 20000 g for 30 minutes at 4 °C for subsequent experimental use.

### Transmission Electron Microscopy

Cultured cells in 35 mm dishes were prepared for TEM via a series of sequential steps. Initial pre‐fixation was performed using a 1:1 mixture of growth medium and 2.5% glutaraldehyde for 5 minutes at room temperature (RT), followed by primary fixation in 2.5% glutaraldehyde in phosphate‐buffered saline (PBS) for 2 hours at RT. After triple washes with PBS, samples underwent ethanol gradient dehydration (50%, 70%, 90%, 95%, and 100% for 8 minutes per concentration), followed by embedding in SPON12 resin. Polymerization was carried out at 60 °C for 48 hours to enable ultrathin sectioning (70 nm) with a diamond knife. Sections were collected on Formvar‐coated 100‐mesh copper grids. After dual staining with uranyl acetate and lead citrate, samples were air‐dried. Final imaging was conducted using a H‐7650B transmission electron microscope with an accelerating voltage of 80 kV.

### Field Emission Scanning Electron Microscopy

Specimens for FESEM were initially fixed in 2.5% glutaraldehyde in PBS at 4 °C overnight, followed by triple PBS washes and secondary fixation with 1% osmium tetroxide containing 1.5% potassium ferrocyanide for 60 minutes at RT. Samples were then subjected to an ethanol gradient dehydration (50% to 100%, 8 minutes per step), followed by substitution with tert‐butanol and cryopreservation at ‐20 °C prior to lyophilization. Sputter‐coating with a 10 nm gold film was performed before ultrastructural analysis using a field emission scanning electron microscope equipped with a secondary electron (SE) detector, under an accelerating voltage of 3 kV.

### Statistical Analysis

All statistical analyses were performed using R software (version 4.2.2) and GraphPad Prism 8.0. Pearson's or Spearman's correlation coefficients were calculated to assess relationships between variables. Data were presented as the mean ± standard deviation (SD) from at least three independent experiments. Statistical significance between two groups was determined using two‐tailed unpaired Student's t‐tests, while one‐way analysis of variance (ANOVA) followed by Tukey's post hoc test was used for multiple group comparisons. A p‐value of less than 0.05 was considered statistically significant (*p < 0.05; ** p < 0.01; *** p < 0.001).

### Ethics Approval Statement

All procedures complied with the Animal Committee of Tongren Hospital guidelines (approval number: A2023‐119‐01). The animal experiments in this study were strictly conducted in accordance with the guidelines provided by the Ministry of Science and Technology of the People's Republic of China regarding the humane treatment of experimental animals, among other regulations

## Conflict of Interest

The authors declare no conflict of interest.

## Author Contributions

K.N.Z., J.C., Z.H.Z., and H.H. contributed equally to this work. L.X., Y.H.W. and Y.Z. designed the study and planned the experiments. K.N.Z., Z.H.Z., J.C. and H.H. performed most of the experiments and bioinformation analysis. R.R.J. and N.W. generated the animal experiments. Q.H.Z. and S.H.X. analyzed the data and performed the statistical analysis. K.N.Z. wrote the manuscript. All authors read and approved the final manuscript.

## Supporting information



Supporting Information

## Data Availability

The data that support the findings of this study are available from the corresponding author upon reasonable request.
