# Supplementary material for: CD74 Blockade Disrupts Endothelial Migrasome Signaling to Prevent Inflammatory Macrophage Differentiation and Inhibit Atherosclerotic Progression
Source: Adv Sci (Weinh). 2025 Jun 23;12(35):e02838. doi: 10.1002/advs.202502838 (PMC12463019; doi:10.1002/advs.202502838)
Supplement: Supplementary file 1 — Supporting Information [file ADVS-12-e02838-s001.docx]

**CD74 Blockade Disrupts Endothelial Migrasome Signaling to Prevent Inflammatory Macrophage Differentiation and Inhibit Atherosclerotic Progression**

Kangnan Zhang, Jiong Chen, Zhenhua Zhu, Hong Hu, Qinghui Zhang, Rongrong Jia, Na Wang, Shihao Xiang, Yong Zhou, Yuehong Wang, Ling Xu

﻿

**Table of contents**

Supplementary figure legends .....................................................................2

Supplementary figures .................................................................................6

Supplementary tables ...................................................................................13

**﻿Supplementary figure legends**

**Figure S1**
**Single-cell analysis of mouse arterial tissue.**A: UMAP plot showing cells from normal and atherosclerotic tissues in the GSE205931 dataset. Five clusters are displayed, each shown in a different color. Batch effects were corrected using the R package Harmony. UMAP analysis was conducted on cells from both normal and atherosclerotic tissues. B: Box plot showing the expression levels of Migrasome_Score in atherosclerotic and normal tissues from the GSE205931 dataset. C: Expression levels of selected known marker genes in unclassified cells from normal and atherosclerotic tissues, visualized on UMAP plots. D: Bubble plot showing the expression of migrasome-related genes in different cell types. The size of the bubbles represents the percentage of cells expressing the gene, while color indicates expression intensity. E: Box plot displaying the expression levels of Migrasome_Score in different cell types. Data information: Data are expressed as mean ± SD. Two-tailed unpaired Student's t-tests.

**Figure S2**
**Single-cell analysis of human atherosclerotic tissue.**A: Dot plot showing the average expression of known markers within each cell cluster. The size of the dots represents the percentage of cells expressing each gene in the cluster, and the intensity of marker expression is displayed. B: Heatmap visualizing cell-cell communication between Endo1-4 cells, T cells, SMC/Fibroblasts, NK cells, neutrophils, Msat cells, and macrophages.

**Figure S3**
**Mechanism of Migrasome Formation in HCAECs Induced by ox-LDL.**

A: Immunofluorescence staining of PIP5K1A and migrasomes in HCAECs. Red: PIP5K1A; Blue: Migrasomes (WGA). (Scale bars: 8 µm). B: Immunofluorescence staining of PIP_2_-RFP and ITGα5-mCherry in HCAECs, with images acquired using confocal microscopy. Red: PIP_2_; Magenta: ITGα5; Blue: Migrasomes (WGA). (Scale bars: 5 µm). C: Immunofluorescence staining of Rab35-GFP and ITGα5-mCherry in HCAECs, with images acquired using confocal microscopy. Green: Rab35; Magenta: ITGα5; Blue: Migrasomes (WGA). (Scale bars: 5 µm). D: RT-qPCR analysis of PIP5K1A and Rab35 mRNA expression levels in vascular endothelial cells transfected with PIP5K1A and Rab35 siRNA or Ctrl, compared to untreated vascular endothelial cells (*n*=3 samples/group). E: Vascular endothelial cells treated with siRNA, stained with 1 µg/ml WGA-Alexa 488 (Scale bars: 10µm). Statistical analysis of migrasome numbers in control and ox-LDL groups, normalized to the control group. Each group includes 50 cells. Data information: Data are expressed as mean ± SD. Two-tailed unpaired Student's t-tests, **p < 0.05; **p < 0.01; ***p < 0.001; ns, not significant.*

**Figure S4**
**Endothelial cells generate migrasomes under ox-LDL stimulation.**A: MAEC and HUVEC cells treated with ox-LDL, stained with 1 µg/ml WGA-Alexa 488 (Scale bars: 10µm), and observed under confocal microscopy and SEM (Scale bars: 1µm). Statistical analysis of migrasome numbers in control and ox-LDL groups, normalized to the control group. Each group includes 50 cells. B: Expression levels of migrasome markers in different MAEC cell lines analyzed by Western blot. The statistical results are shown in a bar chart (*n*=3 samples/group). C: Expression levels of migrasome markers in different HUVEC cell lines analyzed by Western blot. Statistical results are presented in a bar chart (*n*=3 samples/group). D: RT-qPCR analysis of migrasome-related gene mRNA expression in MAEC cells (*n*=3 samples/group). E: RT-qPCR analysis of migrasome-related gene mRNA expression in HUVEC cells (*n*=3 samples/group). Data information: Data are expressed as mean ± SD. Two-tailed unpaired Student's t-tests, **p < 0.05; **p < 0.01; ***p < 0.001; ns, not significant*

**Figure S5**
**Functional Analysis of Endothelial Cells**

A: GO functional analysis of Endo1–4 subclusters. B: Bubble plot showing the expression of PIP5K1A, RAB35, and ITGA5 in Endo1–4. C: GSEA of the “Macrophage activation” pathway. D: GSEA of the “Regulation of macrophage activation” pathway.

**Figure S6**
**Validation of the effect of migrasomes produced by mouse-derived endothelial cells on mouse-derived macrophages.**A: Flow cytometry analysis of macrophage differentiation. B: Statistical bar chart of macrophage differentiation from flow cytometry analysis (*n*=3 samples/group). C: Immunofluorescence analysis of macrophage differentiation. Blue: DAPI; Green: M1 macrophages (Nos2^+^); Red: M2 macrophages (CD206^+^) (Scale bars: 100µm). D: Statistical bar chart of the percentage of fluorescent regions in macrophage differentiation from immunofluorescence analysis (*n*=5 samples/group). E：qPCR analysis of cytokine expression in three groups of cells (n=3 samples/group). Data information: Data are expressed as mean ± SD. One-way analysis of variance (ANOVA) was used followed by Tukey's post hoc test to determine the statistical significance, **p < 0.05; **p < 0.01; ***p < 0.001; ns, not significant.*

**Figure S7**

**Macrophage CD74 blockade and atherosclerosis progression.**A: Expression levels of migrasome-related markers (TSPAN4, TSPAN7, EOGT), CD80, CD206, CD74, and APP were analyzed by Western blot in different groups. Quantitative results from three independent experiments (*n* = 3 per group) are presented in bar graphs. B: Single-cell level analysis of CD74 expression across various cell types within the tissue. C: Comparison of blood pressure (systolic blood pressure, diastolic blood pressure) between the two groups of mice (n=5 mice/group). D: Single-cell suspensions were prepared from arterial tissues and analyzed by flow cytometry. Dead cells were excluded using a viability dye (BV510), followed by sequential gating on CD45⁺ leukocytes and CD11b⁺ myeloid cells. Macrophages were identified as CD45⁺CD11b⁺F4/80⁺ cells (*n*=5 mice/group). E: Comparison of serum lipid levels (TC, TG, HDL-C, and LDL-C) between the two groups of mice (*n*=5 mice/group). F: Comparison of serum inflammatory cytokine levels (LPS, TNF-α, IL-6, and IL-10) between the two groups of mice (*n*=5 mice/group). Data information: Data are expressed as mean ± SD. Two-tailed unpaired Student's t-test, **p < 0.05; **p < 0.01; ***p < 0.001; ns, not significant.*

**
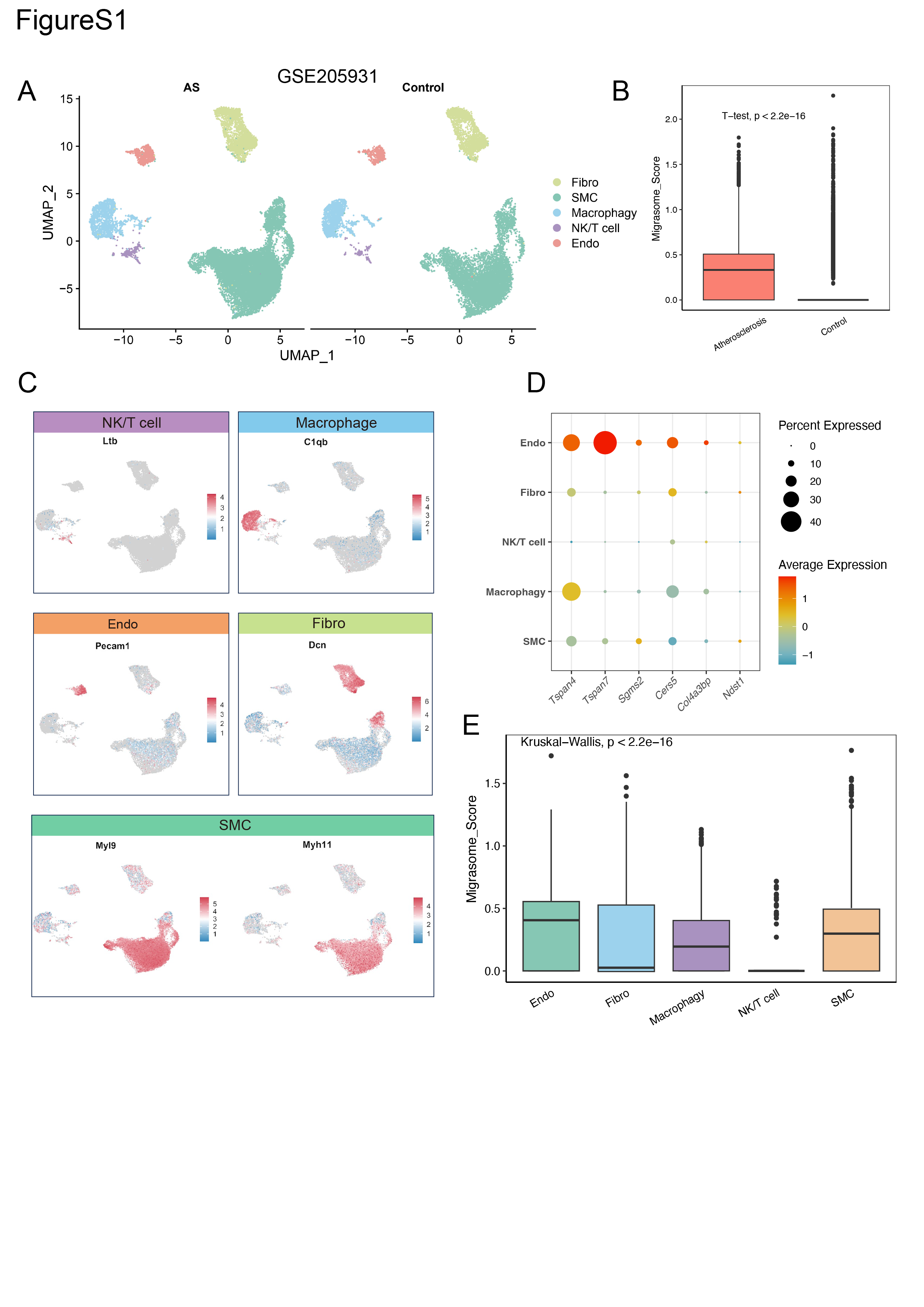
**

**
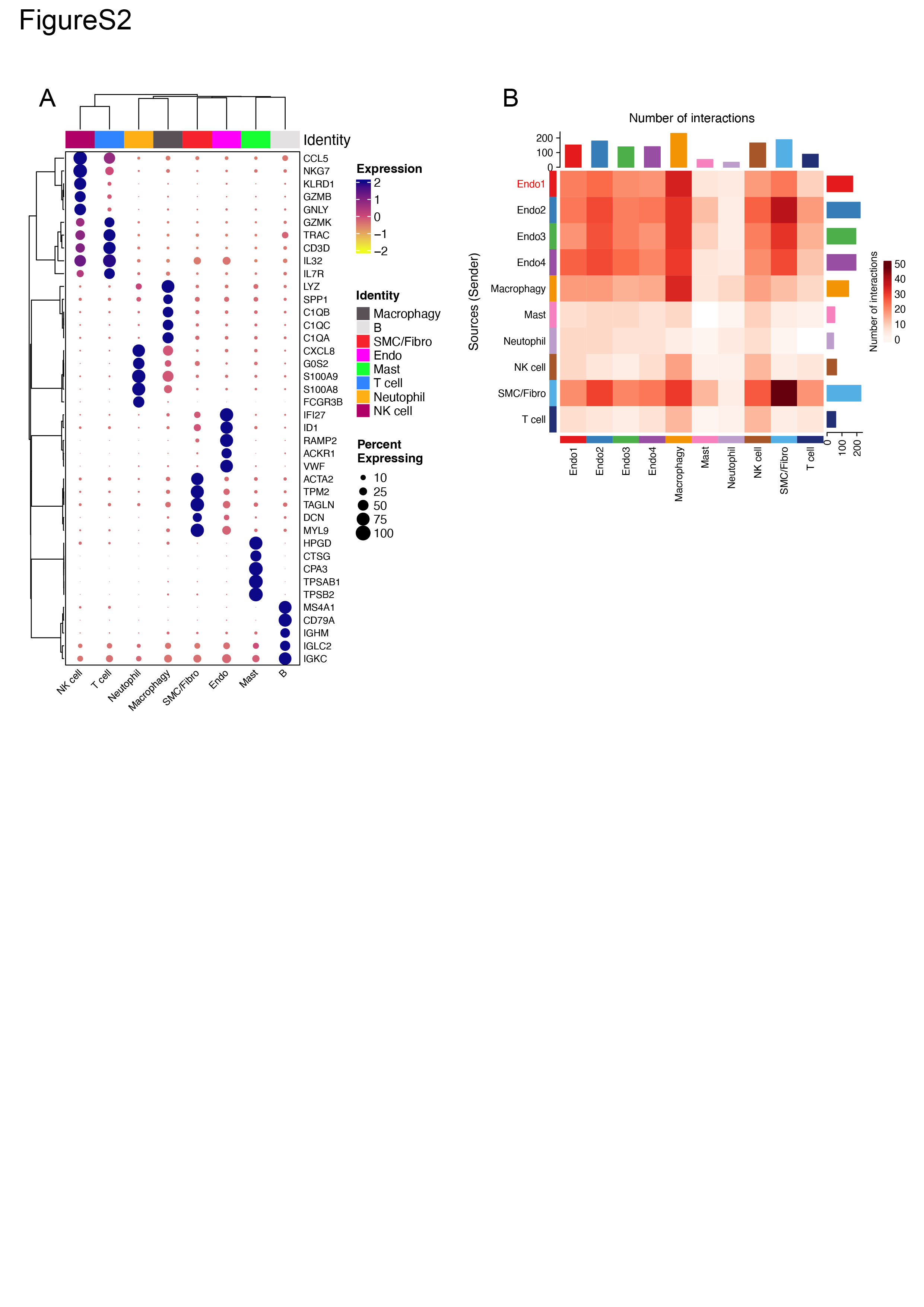
**

**
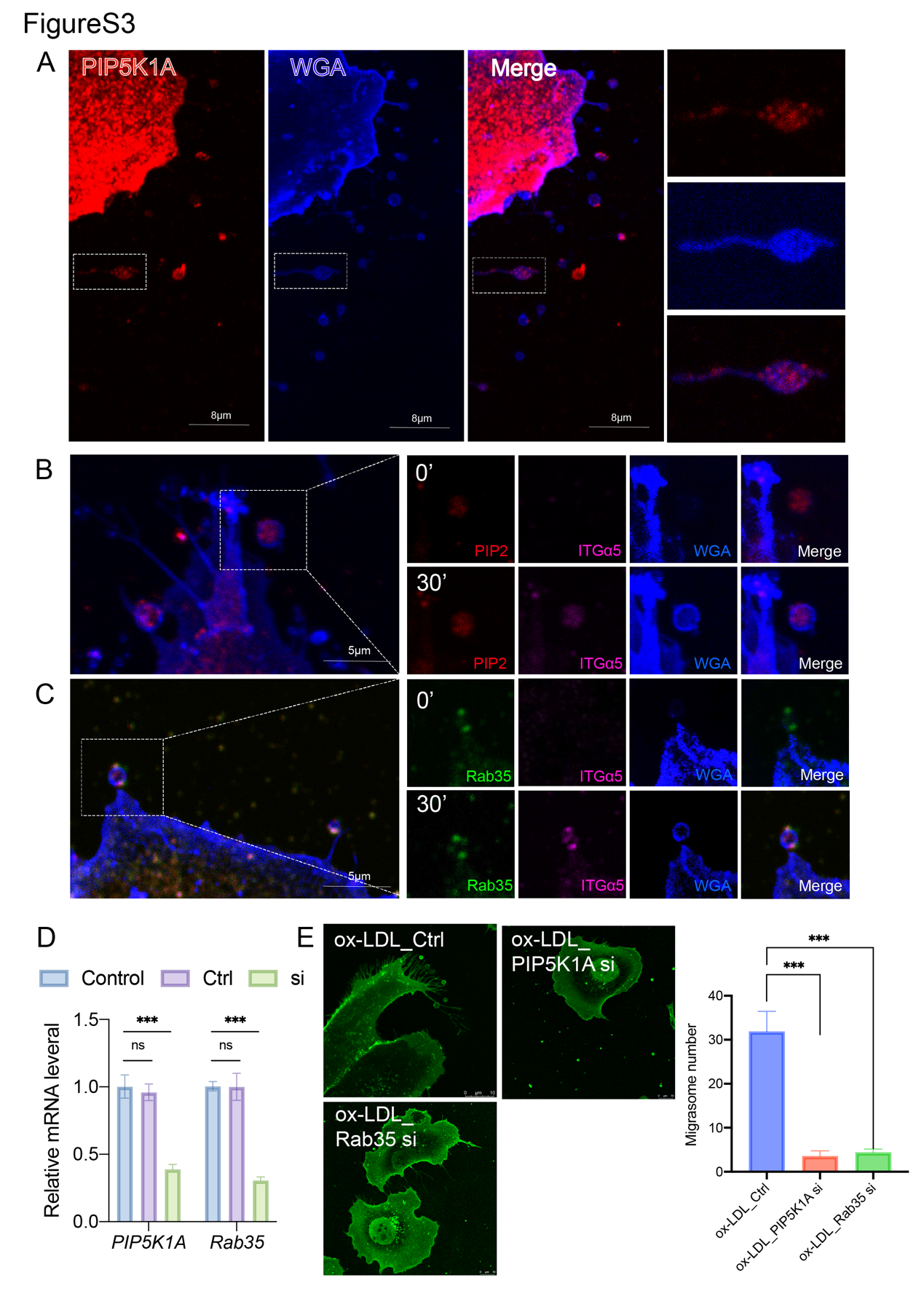
**

**
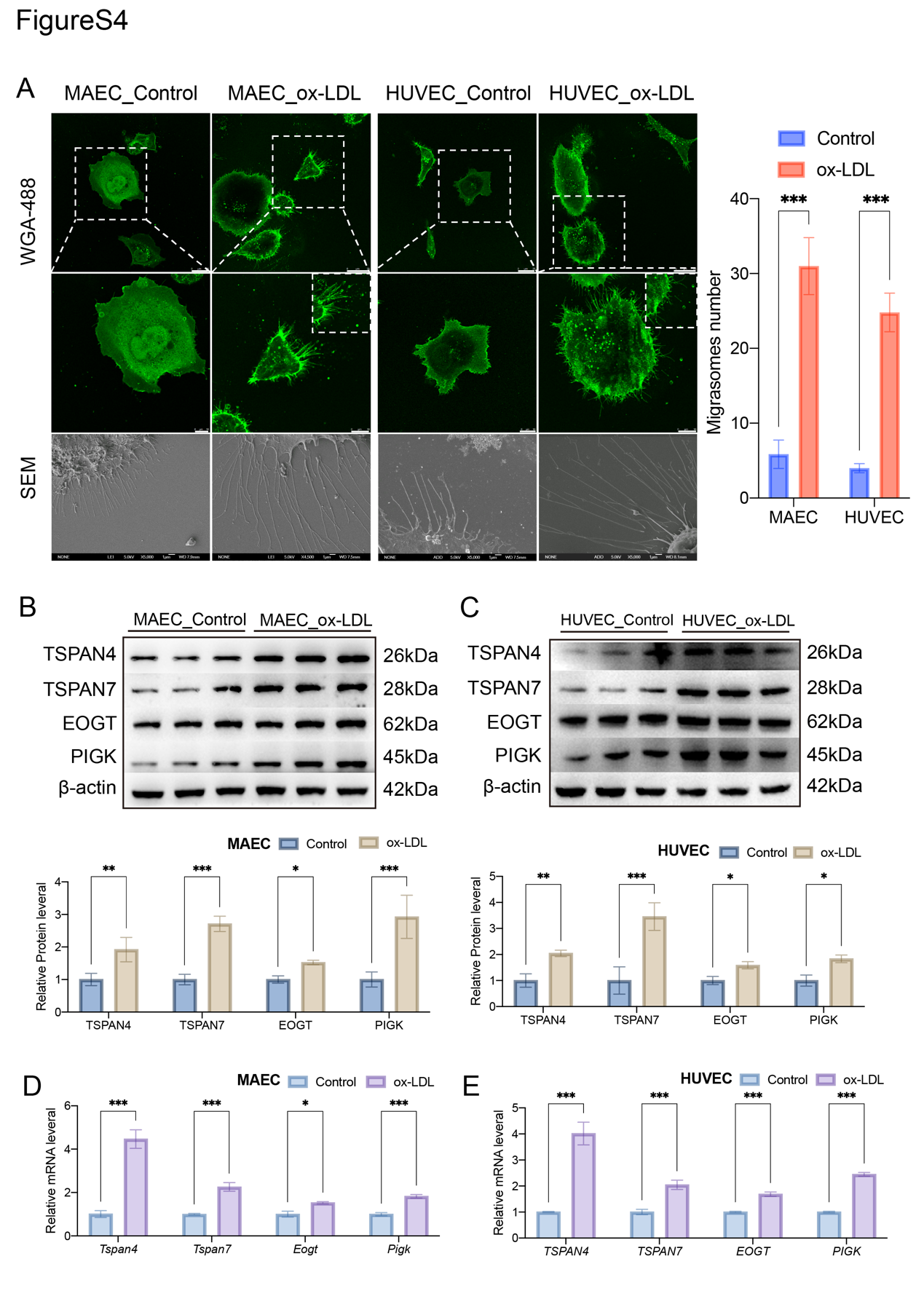
**

**
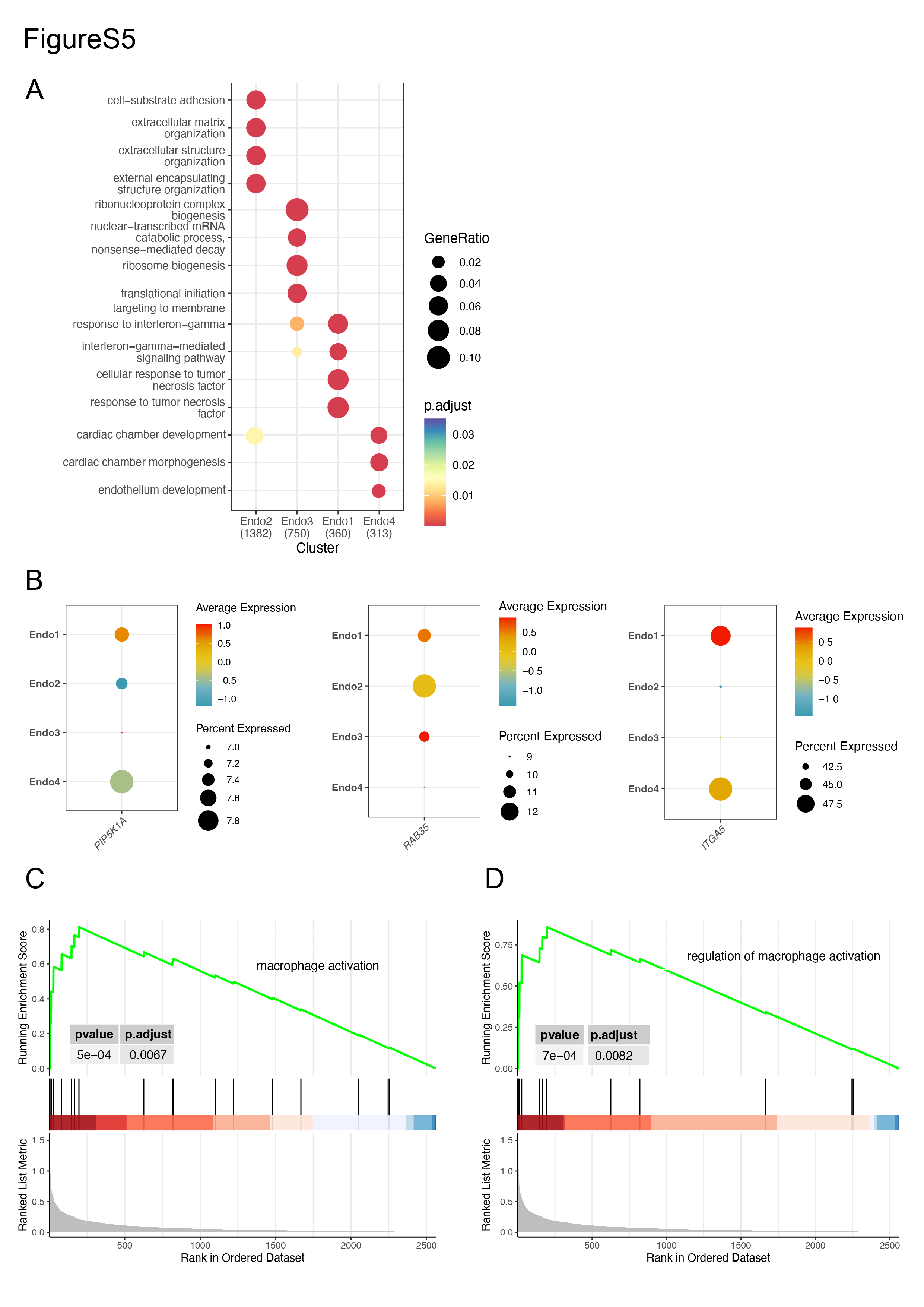
**

**
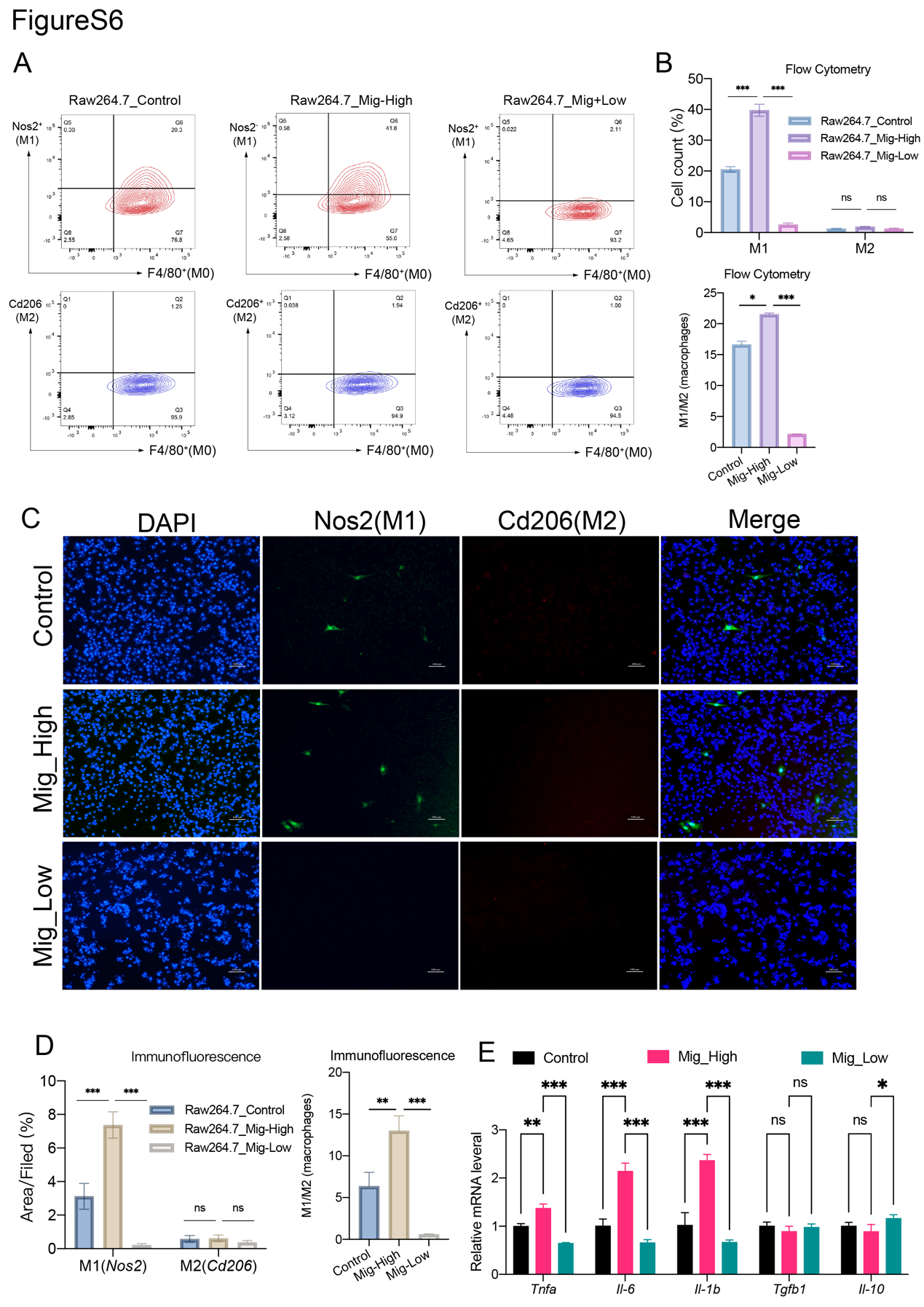
**

**
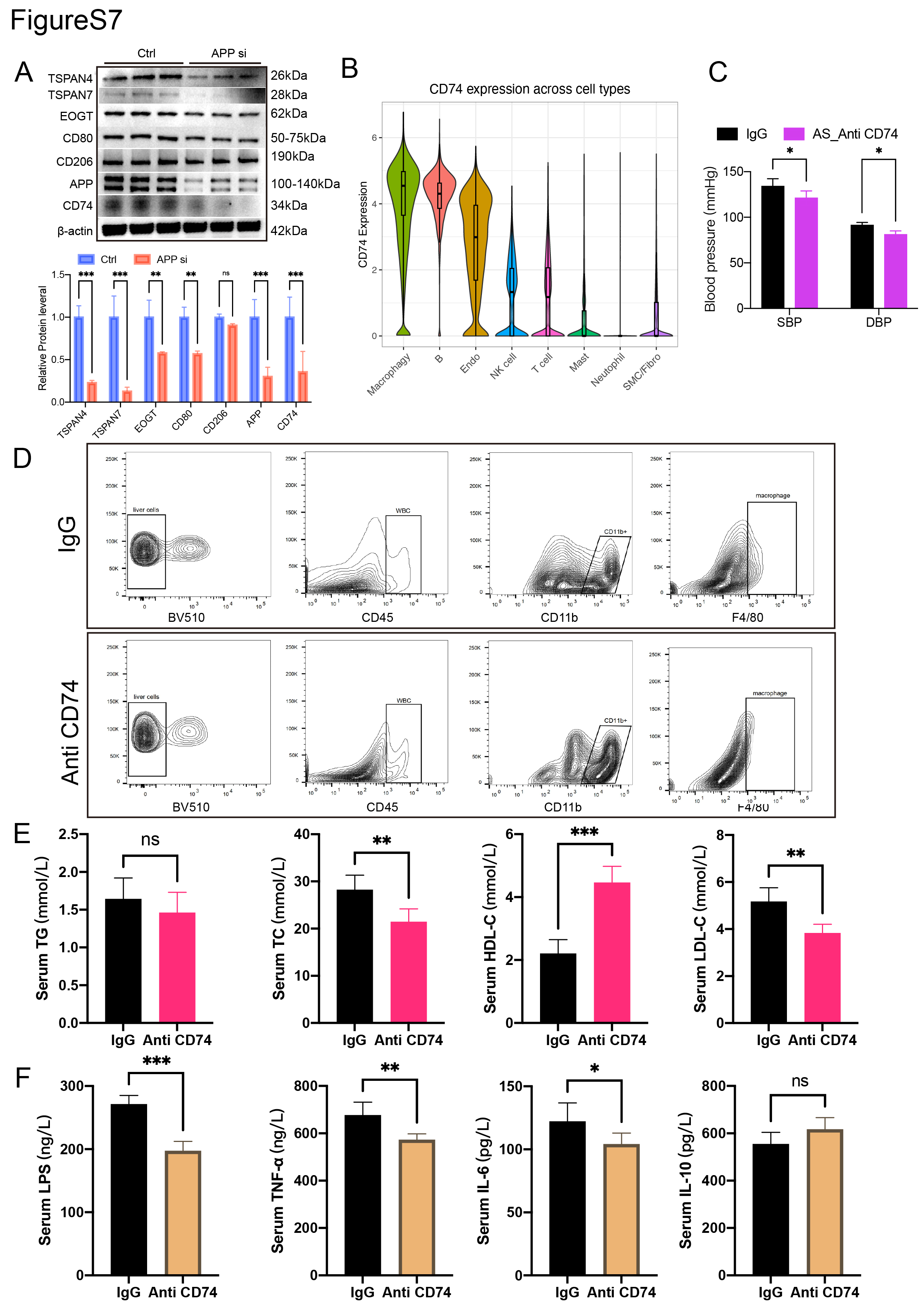
**

**Table S1: Short hairpin RNA (sh-RNA) targeted sequences**

| sh-RNA | Sequences |
| --- | --- |
| TSPAN4-shRNA-human1: | 5′-CGTATTCTCCAAAGCAGTGTT-3′ |
| TSPAN4-shRNA-human2: | 5′-ACGGACAAGATTGACAGGTAT-3′ |
| TSPAN4-shRNA-human3: | 5′-CTCCAACTACACTGACTGGTT-3′ |
| TSPAN4-shRNA-mouse1: | 5′-CAGTGACAAGATTGACAGTTA-3′ |
| TSPAN4-shRNA-mouse2: | 5′-CCCAGGGAACTTTGACACTTT-3′ |
| TSPAN4-shRNA-mouse3: | 5′-GTACCTCATGTTCGCCTTCAA-3′ |

**Table S2: Small interfering (si-RNA) targeted sequences**

| si-RNA | Sequences |
| --- | --- |
| APP-siRNA-human1: | sense-GUUACGGAAACGAUGCUCU |
|  | antisense-AGAGCAUCGUUUCCGUAAC |
| APP-siRNA-human2: | sense-CUACCGCUGCUUAGUUGGU |
|  | antisense-ACCAACUAAGCAGCGGUAG |
| APP-siRNA-human3: | sense-CCGUUGACAAGUAUCUCGA |
|  | antisense-UCGAGAUACUUGUCAACGG |

**Table S3: RT-PCR primer sequences**

| Gene |  | Primer sequences (Human) | Gene |  | Primer sequences (Mouse) |
| --- | --- | --- | --- | --- | --- |
| TSPAN7 | F | ACACGGACGCTATGCAGAC | *Tspan7* | F | AGACCAAACCTGTGATAACCTGT |
|  | R | CCTGGGGATTACAATCAGTTTCG |  | R | GGGAGCATTTGTGGAGTTCTC |
| TSPAN4 | F | GCTGTGGCGTCTCCAACTAC | *Tspan4* | F | TACCTCATGTTCGCCTTCAAC |
|  | R | CTTGGCAGTACATGGTCATGG |  | R | GATAAGGTGGCAAAGTTTCCCT |
| NDST1 | F | GACCTAGCGAGGTGGAGAAAG | *Ndst1* | F | CCACAACTATCACAAAGGCATCG |
|  | R | GGGATGGACTCAGACGAGC |  | R | GAAAGGTGTACTTTAGGGCCAC |
| EOGT | F | GCTCCTCCTAATACTCACAGCA | *Eogt* | F | TTAATGCTGCTTGTCTTTGGAGT |
|  | R | CAGGATTTCTCATAACCCCAGC |  | R | GCAACATGCCTGTTATTGTGC |
| PIGK | F | ACCAACATAGAACTCGCGGAT | *Pigk* | F | GGACACACAAATAACTGGGCT |
|  | R | TCTTCTCCCACTTGACTACTAGC |  | R | GCCTCTTGACGCTTCTATAAACA |
| CPQ | F | GGCTGGAGAAAGTTCACCTGG | *Cpq* | F | CCTATGAGCGTTTGGGACTTC |
|  | R | CCTTCTGGAGGAGTCCCAATG |  | R | GGTGAACATTTTCTAGCCCATCT |
| Actin | F | CATGTACGTTGCTATCCAGGC | *Actin* | F | GTGACGTTGACATCCGTAAAGA |
|  | R | CTCCTTAATGTCACGCACGAT |  | R | GCCGGACTCATCGTACTCC |

**Table S4: RT-PCR primer sequences**

| Cytokine |  | Primer sequences (Human) | Cytokine |  | Primer sequences (Mouse) |
| --- | --- | --- | --- | --- | --- |
| IL-1β | F | AGCTACGAATCTCCGACCAC | *Il-1b* | F | GCAACTGTTCCTGAACTCAACT |
|  | R | CGTTATCCCATGTGTCGAAGAA |  | R | ATCTTTTGGGGTCCGTCAACT |
| IL-6 | F | ACTCACCTCTTCAGAACGAATTG | *IL-6* | F | TAGTCCTTCCTACCCCAATTTCC |
|  | R | CCATCTTTGGAAGGTTCAGGTTG |  | R | TTGGTCCTTAGCCACTCCTTC |
| TNFα | F | GAGGCCAAGCCCTGGTATG | *Tnfa* | F | CCTGTAGCCCACGTCGTAG |
|  | R | CGGGCCGATTGATCTCAGC |  | R | GGGAGTAGACAAGGTACAACCC |
| IL-10 | F | TCAAGGCGCATGTGAACTCC | *Il-10* | F | CTTACTGACTGGCATGAGGATCA |
|  | R | GATGTCAAACTCACTCATGGCT |  | R | GCAGCTCTAGGAGCATGTGG |
| TGFβ | F | CTAATGGTGGAAACCCACAACG | *Tgfb1* | F | CTCCCGTGGCTTCTAGTGC |
|  | R | TATCGCCAGGAATTGTTGCTG |  | R | GCCTTAGTTTGGACAGGATCTG |
| Actin | F | CATGTACGTTGCTATCCAGGC | *Actin* | F | GTGACGTTGACATCCGTAAAGA |
|  | R | CTCCTTAATGTCACGCACGAT |  | R | GCCGGACTCATCGTACTCC |
